# c-Cbl targets PD-1 in immune cells for proteasomal degradation and modulates colorectal tumor growth

**DOI:** 10.1038/s41598-019-56208-1

**Published:** 2019-12-27

**Authors:** Chimera Lyle, Sean Richards, Kei Yasuda, Marc Arthur Napoleon, Joshua Walker, Nkiruka Arinze, Mostafa Belghasem, Irva Vellard, Wenqing Yin, Jonathan D. Ravid, Elias Zavaro, Razie Amraei, Jean Francis, Uma Phatak, Ian R. Rifkin, Nader Rahimi, Vipul C. Chitalia

**Affiliations:** 1 Department of Medicine, Boston University School of Medicine, Boston, MA USA; 2 Department of Surgery, Boston University School of Medicine, Boston, MA USA; 3 Department of Pathology and Laboratory Medicine, Boston University School of Medicine, Boston, MA USA; 4 School of Medicine, Boston University, Boston, MA USA; 5 Veterans Affairs Boston Healthcare System, Boston, MA USA; 6 Global co-creation Labs, Institute of Medical Engineering and Sciences, Massachusetts Institute of Technology, Boston, MA USA

**Keywords:** Cancer models, Colon cancer

## Abstract

Casitas B lymphoma (c-Cbl) is an E3 ubiquitin ligase and a negative regulator of colorectal cancer (CRC). Despite its high expression in immune cells, the effect of c-Cbl on the tumor microenvironment remains poorly understood. Here we demonstrate that c-Cbl alters the tumor microenvironment and suppresses Programmed cell death-1 (PD-1) protein, an immune checkpoint receptor. Using syngeneic CRC xenografts, we observed significantly higher growth of xenografts and infiltrating immune cells in c-Cbl^+/−^ compared to c-Cbl^**+/+**^ mice. Tumor-associated CD8+ T-lymphocytes and macrophages of c-Cbl^+/−^ mice showed 2–3-fold higher levels of PD-1. Functionally, macrophages from c-Cbl^+/−^ mice showed a 4–5-fold reduction in tumor phagocytosis, which was restored with an anti-PD-1 neutralizing antibody suggesting regulation of PD-1 by c-Cbl. Further mechanistic probing revealed that C-terminus of c-Cbl interacted with the cytoplasmic tail of PD-1. c-Cbl destabilized PD-1 through ubiquitination- proteasomal degradation depending on c-Cbl’s RING finger function. This data demonstrates c-Cbl as an E3 ligase of PD-1 and a regulator of tumor microenvironment, both of which were unrecognized components of its tumor suppressive activity. Advancing immune checkpoint and c-Cbl biology, our study prompts for probing of PD-1 regulation by c-Cbl in conditions driven by immune checkpoint abnormalities such as cancers and autoimmune diseases.

## Introduction

A recent analysis of 490,305 patients showed that adults born circa 1990, compared with those born circa 1950, have double the risk of colon cancer and quadruple the risk of rectal cancer^1^. One in 23 women and 1 in 21 men in the United States will develop sporadic colorectal cancer (CRC) in their lifetime. Underlying 90% of sporadic CRC patients is the loss-of-function mutation of *Adenomatosis Polyposis Coli* that drive aberrant activation of the oncogenic Wnt/β-catenin pathway in colonic epithelium^2^.

Casitas B-lineage lymphoma (c-Cbl) is a RING-domain containing E3 ubiquitin ligase extensively studied in myeloid cells and myeloid malignancies^3,4^. c-Cbl knock-out mice present with an immune phenotype characterized by splenomegaly and alterations in positive thymic selection^5^. Recent studies in mouse models and human CRC tumors showed c-Cbl as a ubiquitin ligase of nuclear β-catenin and a regulator of angiogenesis and tumorigenesis through different mechanisms^6–10^. The expression of c-Cbl in human CRC tumors inversely correlated with nuclear β-catenin and the overall survival of patients with metastatic CRC^9–11^. Xenograft studies demonstrated that CRC cells silenced for *c*-*Cbl* showed augmented tumor growth. However, the studies performed in nude mice with immunocompromised background precluded the examination of tumor microenvironment. The tumor microenvironment is a critical regulator of tumor growth and is likely to be altered by c-Cbl given its high expression in myeloid and lymphoid cells^3,4,12^.

We set out to examine the tumor microenvironment upon c-Cbl modulation. Our results demonstrated that the loss of c-Cbl activity resulted in rapid growth of tumor and increased expression of programmed cell death (PD-1) receptors in tumor infiltrating CD8+ T-lymphocytes and macrophages. PD-1 is an immune checkpoint protein and its expression inhibits immune cells in the tumor microenvironment to augment tumor growth^13^. Previous studies have focused on CD8+ T-lymphocytes in which the interruption of PD-1 with its cognate ligands PDL-1 and PDL-2 resulted in tumor regression^13^. Recent reports have uncovered the importance of PD-1 in tumor associated macrophages (TAM) and showed that bone marrow-derived macrophages (BMDM) homing to the tumor microenvironment expressed increasing amounts of PD-1 in mouse models of CRC and in human CRC^14^. Increase in PD-1 expression levels on TAM inhibited tumor phagocytosis to augment tumor growth. Despite its profound translational significance, the basic biology of PD-1 such as its degradation remains to be established.

## Results

**Figure 1:**
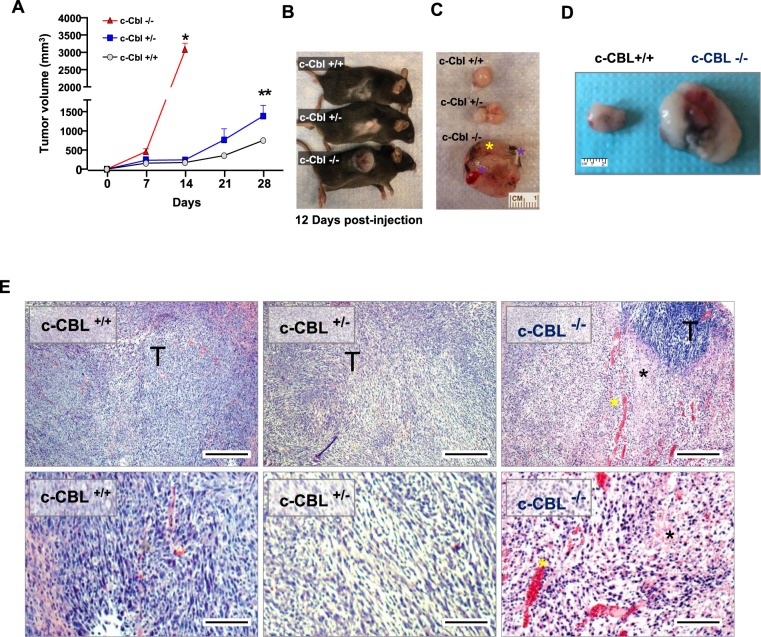
Reduced c-Cbl activity enhances tumor growth and immune infiltrates. (**A**) Average of tumor volumes from six mice injected subcutaneously with MC38 cells in c-Cbl^+/+^ and c-Cbl^+/−^ groups and five in the c-Cbl^−/−^ group. All the c-Cbl^−/−^ mice had to be euthanized by 10–12 days with rapid development of the xenograft along with ulceration on the tumor. Student’s t-test was performed. Error bars = SEM. *p < 0.0001 corresponds to growth of tumors in c-Cbl^−/−^ mice compared to both c-Cbl^+/−^ and c-Cbl^+/+^ groups. **p = 0.003 compares the group tumor growth c-between Cbl^+/−^ and c-Cbl^−/−^ group. (**B**) A representative mouse from three groups is shown at 12 days post-injection. (**C**) Excised xenografts are shown. Blue asterisk corresponds to the areas of necrosis and liquefaction (soft by palpation) compared to other areas of xenografts and yellow asterisk demarcates areas of ulceration. (**D**) Excised MC38 xenografts are shown. Representative images of a pair of c-Cbl^+/+^ and c-Cbl^+/−^ mice are shown. (**E**) H&E stain of xenografts from different groups of mice are shown at 40X and 100X magnification. Xenografts from c-Cbl^+/+^ and c-Cbl^+/−^ mice showed tumor cells (T) without evidence of acellular areas with fragmented nuclei suggestive of necrosis. Xenografts from c-Cbl^−/−^ mice showed acellular areas suggestive of necrosis (marked with a black asterisk) and several areas of pools of RBCs suggestive of hemorrhage (marked with a yellow asterisk). Scale bar = 25 micron (40×) and 50 micron (100×).

The status of c-Cbl kockout in mice was confirmed by genotyping (Supplementary Fig. 1A)^5^. Given high expression of c-Cbl in immune cells, spleen was examined for CBL mRNA and c-Cbl protein. Extracts from spleen showed a significant reduction in *CBL* mRNA and c-Cbl protein levels corresponding to the status of *CBL* gene (Supplementary Fig. 1A). Next, we examined effects of c-Cbl reduction on tumor microenvironment using a syngeneic xenograft model; MC38 colon adenocarcinoma derived from C57BL/6 mice (a background similar to that of c-Cbl^+/−^ mice). Compared to the c-Cbl^+/+^ mice (750 + 141.14 mm^3^), MC38 xenografts in c-Cbl^+/−^ mice showed a higher growth of the tumor and had double volume (average + SD 1290 + 251.9 mm^3^, p = 0.003) by the end of 4 weeks (Fig. 1A–D). c-Cbl^−/−^ showed an even faster growth of xenograft. Within 12 days of post-injection, MC38 xenografts of c-Cbl^−/−^ mice reached a significantly higher volume (3052 + 209.5 mm^3^, p < 0.001) compared to other groups (c-Cbl^+/+^: 156 + 10 mm^3^ and c-Cbl^+/−^: 202 + 35 mm^3^) (Fig. 1A). Xenografts of c-Cbl^−/−^ mice showed extensive skin ulceration warranting early euthanasia (Fig. 1B–D). Histopathology examination of xenografts of c-Cbl^+/−^ and c-Cbl ^+/+^ mice showed tumor cells. However, the xenograft from c-Cbl^−/−^ mice revealed areas of severe necrosis and hemorrhage interspersed amidst the tumor cells (Fig. 1E). Since these changes in the xenografts will alter the microenvironment, subsequent studies were conducted in c-Cbl^+/+^and c-Cbl^+/−^ mice.

**Figure 2:**
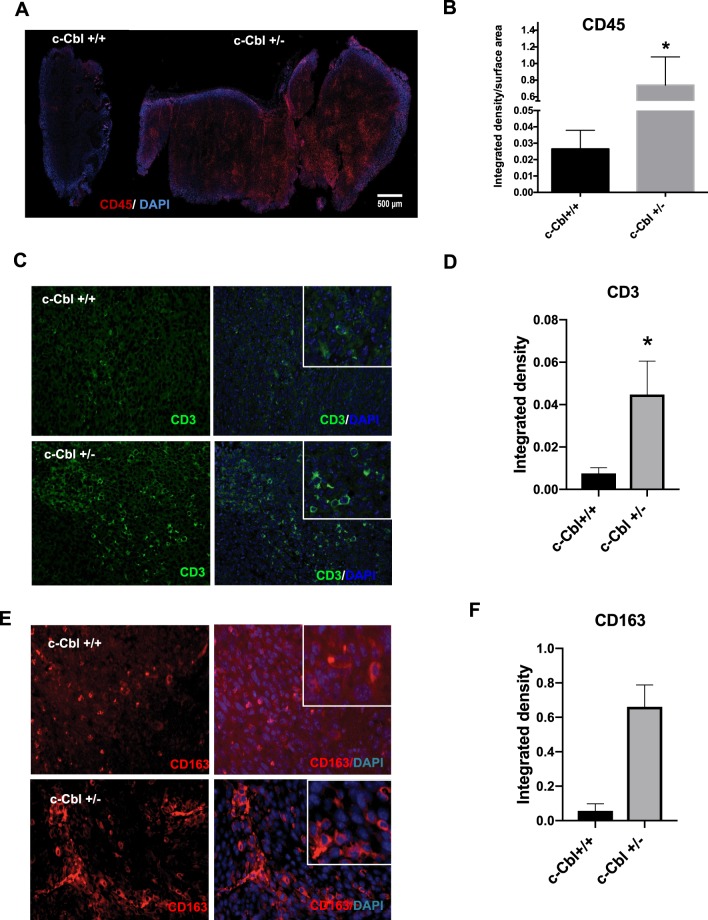
Increased immune infiltrate in xenografts of c-Cbl^+/−^ mice. (**A**) Xenografts were stained for CD45+ cells and DAPI for the nuclei. Intense DAPI stain at the periphery of the tumor is due to increase staining at the edges (edge effect). Representative Images from six xenografts per group. (**B**) CD45+ cell infiltration was quantitated as integrated density and normalized for the area of the tumor in square micron. Average of four sections of the tumor per mouse is shown. Total of six tumors per group. Student’s t-test was performed. Error bars = SEM. p = 0.043. (**C**) Xenograft from each mouse was stained for CD3+ cells. The insert shows tumor-associated lymphocytes. DAPI stained nuclei. Representative images from six xenografts per group are shown. (**D**) CD3+ cell infiltration was quantitated as integrated density. Average of ten sections of the tumor per mouse is shown. Total of six tumors per group. Student’s t-test was performed. Error bars = SEM. p = 0.03. (**E**) Xenograft from each mouse was stained for CD163+ cells. The insert shows the tumor-associated macrophages. Representative images from six xenografts per group are shown. (**F**) CD163+ cell infiltration was quantitated as integrated density. Average of ten sections of the tumor per mouse is shown. Total of six tumors per group. Student’s t-test was performed. Error bars = SEM. P < 0.001.

To further evaluate the tumor microenvironment, the xenografts were probed using a CD45+ antibody, a maker present on all leucocytes. Xenografts of MC38 from c-Cbl^+/−^ mice showed more CD45+ infiltrates in comparison to c-Cbl^+/+^ mice (Fig. 2A), which was quantitated as the intensity density, a composite of pixel intensity and number, normalized to the tumor area. The tumor sections from c-Cbl^+/−^ mice showed a significantly higher intensity density (0.73 + 0.34 intensity density/micron^2^) compared to c-Cbl^+/+^ mice (0.02 + 0.01 intensity density/micron^2^, p = 0.043) (Fig. 2B). These immune infiltrates consisted of both CD3+ lymphocytes and monocyte/macrophage lineage CD163+ cells (Fig. 2C–F). The quantification of both these immune infiltrates revealed a higher number of lymphocytes and a significantly higher number of macrophages in xenografts from c-Cbl^+/−^ mice. These data suggested that the haploinsufficiency of c-Cbl increased tumor growth and immune infiltrates in the xenografts.

c-Cbl is highly expressed in myeloid cells^4^ and is involved in macrophage phagocytosis^15^. Upon infiltration in tumors, BMDM are known to phagocytose the tumor cells thereby assisting in regulating tumor growth^14,16^. Based on this previous work, we anticipated that the increased macrophage infiltrates in c-Cbl^+/−^ xenografts are likely to result in a lower tumor growth. Interestingly, our results were contrary to this expectation (Fig. 1D,H). We therefore posited that if c-Cbl^+/−^ macrophages have reduced ability to phagocytose tumor cells. This contention was examined in BMDMs co-cultured with GFP-labeled tumor cells. Percentage of GFP+ macrophages served as a readout of tumor phagocytosis. Phagocytosis performed at 4 °C served as controls. Tumor cell phagocytosis by c-Cbl^+/−^ macrophages was lower (6.5 + 1.59%) compared to that of c-Cbl^+/+^ macrophages (20.13 + 3.41%, p = 0.03) (Supplementary Fig. 2A,B). Tumor phagocytosis at 4^0^c was the lowest. These data suggested that reduction in c-Cbl activity compromised the ability of macrophages to phagocytose tumor cells.

**Figure 3:**
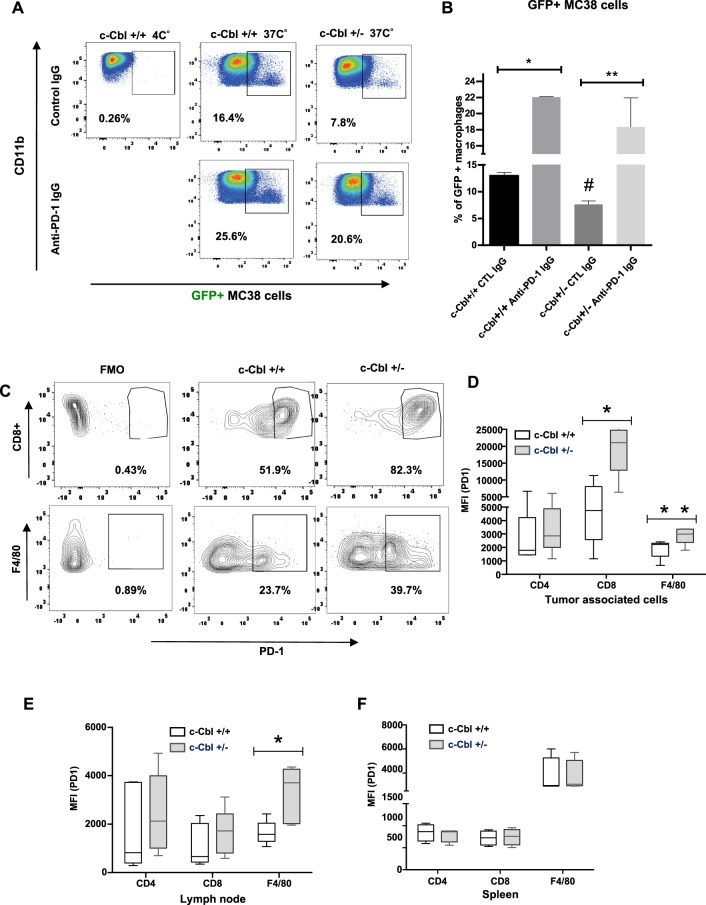
Reduced c-Cbl activity compromises tumor phagocytosis while increasing PD-1 expression in macrophages. (**A**) BMDM differentiated from c-Cbl^+/+^ and c-Cbl^+/−^ for 7 days were co-cultured with GFP+ MC38 tumor cells for 4 hours and treated with iso-type control or anti-PD-1 neutralizing antibodies. Representative FACS from three independent experiments is shown. Gate set with GFP and Fluorescence Minus One (FMO) served as control. (**B**) Averages from three independent experiments with MC38 cells are shown. 2-way ANOVA and Student’s t-test were performed. Error bars = SEM. ^#^p = 0.013 compares c-Cbl^+/+^ and c-Cbl^+/−^ macrophages treated with the control antibody, *p = 0.002, **p = 0.05. (**C**) MC38 xenografts were harvested and the tumor-associated immune cells were sorted for live and dead cells using Zombie dye followed by cell-type specific markers such as CD8 for cytotoxic T-lymphocytes and F4/80 for activated macrophages. The Fluorescence Minus One Control or FMO served as control. Representative FACS plots from six independent tumors are shown. Gates set with PD-1 Fluorescence Minus One (FMO) control. (**D**) Mean fluorescence intensity (MFI) of PD-1 on tumor-associated immune cells from six mice in each group is shown as box plots. The borders of the box depict 25th and 75th percentile and whisker corresponding to minimum and maximum values. Student’s t-test were performed. *p = 0.007, **p = 0.050. (**E**) MFI of PD-1 on cells from lymph nodes of tumor-injected six mice in each group is shown. Student’s t-test were performed. * p = 0.002. (**F**) MFI of PD-1 from splenic cells from un-injected mice six mice per group. Error bars = SEM.

Recent studies have shown that macrophages perform tumor phagocytosis in a PD-1 dependent manner^14,17,18^. In these studies, treatment with pre-validated anti-PD-1 neutralizing antibody strikingly increased tumor phagocytosis by macrophages. In light of these findings, we examined tumor phagocytosis by Cbl^+/−^ macrophages pre-treated with anti-PD-1 neutralizing antibody followed by exposure to GFP+ MC38 tumor cells (Fig. 3A). Using isotype antibody (serving as a control) and GFP+ MC38 tumor lines, at 37 °C, the Cbl^+/−^ macrophages showed a significant reduction in phagocytosis (7.63 + 0.14%) compared to Cbl^+/+^ macrophages (12.95 + 1.68%, p = 0.013) (Fig. 3B).

Treatment of Cbl^+/+^ macrophages with anti-PD-1 neutralizing antibody prior to phagocytosis of GFP+ MC38 cells increased the % of GFP+ macrophages from 12.95 + 1.68% (control antibody) to 22.05 + 1.68% (p = 0.002), which corresponds to approximately 76% increase in phagocytosis (Fig. 3A,B). While a similar treatment of Cbl^+/−^ macrophages increased tumor phagocytosis by 2.3-fold (control antibody 7.63 + 0.14%, anti-PD-1 neutralizing antibody 18.35 + 2.15%, p = 0.05). In fact, it restored tumor phagocytosis to a level similar to Cbl^+/+^ macrophages (p = 0.158). The above results were further supported by an orthogonal assay to detect macrophage phagocytosis. BMDM from c-Cbl^+/+^ and c-Cbl^+/−^ mice pre-treated with anti PD-1 neutralizing antibody or isotype control were co-cultured with GFP+ MC38 cells and subjected to confocal microscopy. BMDMs were stained with PD-1 antibody and phagocytosed MC38 cells were detected by intracellular GFP signal. The PD-1+ cells with GFP+ were counted and presented as the percentage of total PD-1+ cells. The GFP+ cells outside/adjacent to the macrophages were excluded from the analysis (Supplementary Fig. 2C, right upper panel, marked with a white # symbol). PD-1 staining in macrophages was observed in the membrane and the cytosol as the cells were permeabilized. Our analysis revealed that 10.40% + 1.51% (mean + SD) of c-Cbl^+/+^ macrophages treated with control IgG were GFP+. This proportion increased to 15.6% + 4.03% with the anti-PD-1 neutralizing antibody (p = 0.027). The c-Cbl^+/−^ macrophages had significantly lower GFP+ cells, 4.60% + 2.96% compared to c-Cbl^+/+^ macrophages (^#^p = 0.004, Supplementary Fig. 2D). Treatment with anti-PD-1 neutralizing antibody increased the number of GFP+ c-Cbl^+/−^ macrophages by 4-fold (18.60% + 4.72%, p = 0.005). These results demonstrated that c-Cbl^+/−^ macrophages had reduced ability for tumor phagocytosis, which was rescued with anti-PD-1 neutralizing antibody. Taken together, both the above-described assays suggested that c-Cbl regulates tumor cell phagocytosis in a PD-1 dependent manner.

**Figure 4:**
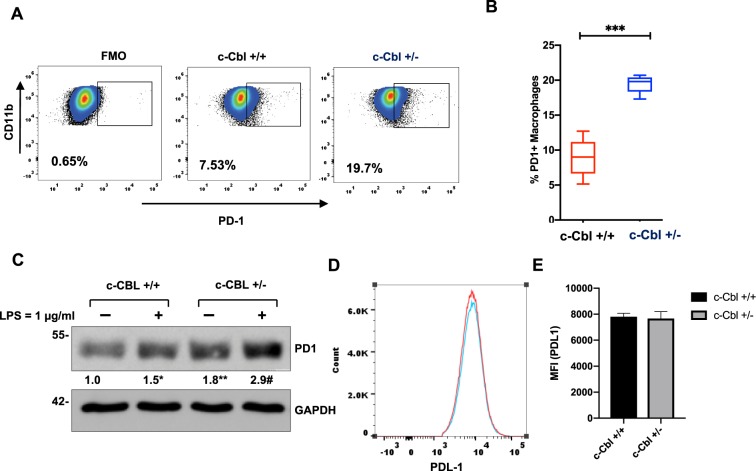
Bone marrow derived c-Cbl^+/−^ macrophages express higher levels of PD-1. (**A**) BMDM differentiated from c-Cbl^+/+^ and c-Cbl^+/−^ for 7 days were subjected to FACS analysis. The cells were gated using CD11b and PD-1. Percentage of PD-1+ macrophages are shown. Fluorescence minus one (FMO) served as a control. Representative FACS plots from three independent experiments are shown. (**B**) Average from three independent experiments is shown. Student’s t-test were performed. Error bars = SEM. *p < 0.001. (**C**) Lysates of BMDMs from mice differentiated for 7 days and treated with LPS overnight. Representative immunoblots from three independent experiments. Student’s t-test with Bonferroni correction was performed. *p = 0.004 for PD-1 in c-Cbl^+/+^ macrophages with and without LPS, **p = 0.001 for PD-1 between c-Cbl^+/+^ and c-Cbl^+/−^ macrophages without LPS and ^#^p = 0.02 for LPS-treated c-Cbl^+/+^ and c-Cbl^+/−^ macrophages. Here, and in all other blots, molecular weights in kilo Dalton are denoted on the left. (**D**) BMDM from c-Cbl^+/+^ (red line) and c-Cbl^+/−^ (blue line) mice differentiated for 7 days and treated with LPS overnight were examined using FACS for PDL-1. Representative MFI of PDL-1 from three independent pairs of mice is shown. (**E**) Average MFI of PDL-1 on BMDM obtained from both the groups (N = 3 mice/group) is shown. Student’s t-test were performed. Error bars = SEM. p = 0.3.

These results raised a possibility of the alterations of PD-1 levels in Cbl^+/−^ macrophages, which was further examined in both TAMs (Fig. 3) and BMDMs (Fig. 4). For TAMs, we used F4/80, a marker of activated macrophages. Analysis of PD-1+ TAMs in MC38 xenografts of c-Cbl^+/−^ showed a higher number of F4/80+ PD-1+ cells (average + SD 37.3 + 9.2%) compared to c-Cbl^**+/+**^ xenografts (21.2 + 4.55, p = 0.048) (Fig. 3C). The mean fluorescence intensity (MFI) of PD-1 in TAMs of c-Cbl^+/−^ mice was 2889 + 285 compared to 1911 + 315 in c-Cbl^+/+^ mice (p = 0.043) (Fig. 3D). Similarly, CD8+ T-lymphocytes from c-Cbl^+/−^ mice xenografts showed close to a 4-fold increase in the MFI of PD-1 (average + SD 19308 + 3594) compared to c-Cbl^+/+^ mice (5195 + 1668, p = 0.007) and higher % of PD-1+ CD8+ T-lymphocytes in c-Cbl^+/−^ mice (76.05 + 8.12%) compared to c-Cbl^+/+^ mice (41.03 + 12.89%, p = 0.052) (Fig. 3C). No changes in MFI of PD-1 were noted in CD4+ T-lymphocytes in tumors. Tumor-draining and other lymph nodes showed doubling of PD-1 expression on macrophages (c-Cbl^+/−^ 3262 + 515 v/s c-Cbl^**+/+**^ 1646 + 218, p = 0.020) (Fig. 3E). On the other hand, immune cells from the spleen of c-Cbl^+/−^ and c-Cbl^**+/+**^ mice showed no differences in macrophages and on CD8+ T-lymphocytes PD-1 MFI (Fig. 3F). However, PD-1 expression is known to be low in spleen macrophages^19^. Together, these results showed higher PD-1 levels in tumor-associated immune cells from c-Cbl^+/−^ mice.

We next examined the expression of PD-1 in macrophages from mice at baseline (not injected with tumors). Fluorescence activated cell sorting (FACS) analysis of BMDM from c-Cbl^+/−^ mice showed more than two-fold greater percentage of PD-1+ macrophages (c-Cbl^**+/+**^ 8.91 + 2.75%, c-Cbl^+/−^ 19.46 + 1.2%, p < 0.001) (Fig. 4A,B). BMDM were lyzed to examine the expression of c-Cbl and PD-1 in whole cell lysates. Compared to c-Cbl^**+/+**^ macrophages, c-Cbl^+/−^ macrophages had 55% reduction in c-Cbl expression (Supplementary Fig. 2E) and a significant (65–80%) increase in PD-1 expression (Fig. 4C). PD-1 expression was further augmented with LPS. Collectively, the above results suggested that Cbl^+/−^ mice had higher surface PD-1 at baseline and in tumor associated macrophages and CD8+ lymphocytes.

The restoration of phagocytosis in c-Cbl^+/−^ macrophages using anti-PD-1 neutralizing antibody can potentially be due to an effect on PDL-1, a ligand of PD-1 receptor. A previous study showed that c-Cbl regulated PDL-1 through STAT, ERK and AKT signaling in lung cancer cells^20^. TAMs are also known to express PDL-1^21^. Therefore, we examined if c-Cbl activity regulated PDL-1 expression on macrophages. Our FACS analysis showed no difference in the levels of PDL-1 in BMDM from c-Cbl^+/−^ and c-Cbl^**+/+**^ mice (p = 0.04) (Fig. 4D,E).

**Figure 5:**
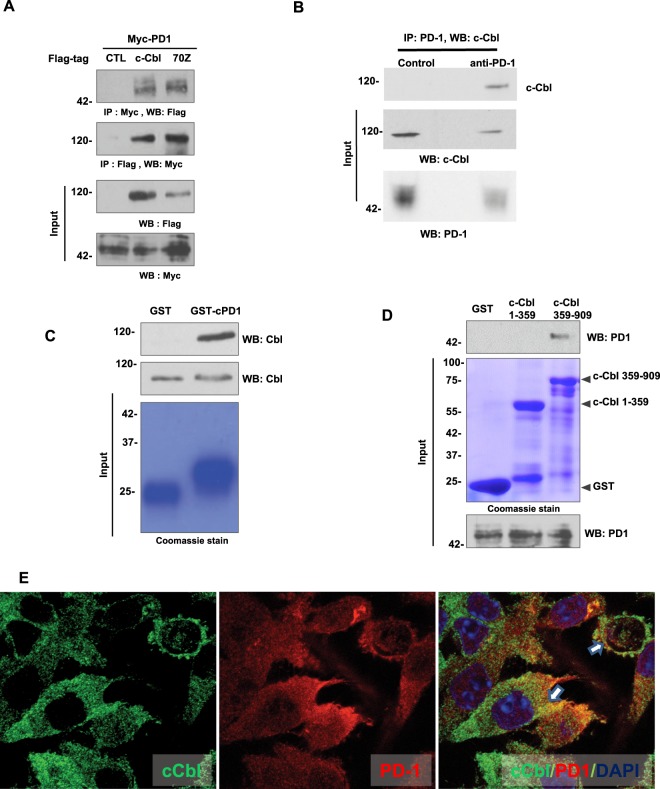
Cytoplasmic tail of PD-1 interacts with the C-terminus of c-Cbl to suppress PD-1. (**A**) Lysates of HEK293T cells co-expressing Myc-tagged PD-1 and Flag-tagged-c-Cbl or 70Z-c-Cbl were immunoprecipitated with Myc-tagged antibody and probed using Flag-tag antibody and vice versa. Five percent of inputs were probed separately. Representative images of three independent experiment are shown. (**B**) Lysates of RAW264.7 macrophages cells were immunoprecipitated with PD-1 antibody and probed using c-Cbl antibody. Five percent of inputs were probed separately. Representative images of two independent experiments are shown. (**C**) Recombinant GST-tagged intracytoplasmic tail of PD-1 was treated with lysates from RAW267.4. The eluents were probed for c-Cbl. Inputs were probed separately. The GST-beads were stained using Commassie dye. Representative immunoblots of three experiments are shown. (**D**) Recombinant GST-tagged c-Cbl 1-359 and 359–909 were treated with lysates from RAW264.7 cells. The eluents were probed for PD-1. 5% of lysates and the beads stained with Commassie are shown as inputs. Representative immunoblots of three experiments are shown. (**E**) Laser confocal microscopy was performed on RAW264.7 cells fixed and stained for c-Cbl and PD-1. Representative images from 100 randomly counted cells are shown. Scale bar = 10 μM.

All of the above data raised a possibility of regulation of PD-1 by c-Cbl. First, we examined their interaction using a reciprocal immunoprecipitation assay performed on extracts of HEK293T cells co-expressing Myc-tagged PD-1 and Flag-tagged c-Cbl followed by immunoprecipitation using Flag-tagged or Myc-tagged antibody. The immunoprecipitation of c-Cbl showed co-precipitation of PD-1 and vice versa (Fig. 5A). This interaction was also observed in RAW264.7 macrophages where the endogenous c-Cbl interacted with the endogenous PD-1. Immunoprecipitation of PD-1 showed co-immunoprecipitation of c-Cbl in these cells (Fig. 5B). Both these data supported an interaction between PD-1 and c-Cbl in different cell types. In addition to wild-type c-Cbl, we used 70Z-c-Cbl (a naturally-occurring mutant of c-Cbl) is known to bind to but fails to degrade other targets of c-Cbl; thus, serving as a dominant negative^8,11^. 70Z-c-Cbl did show binding to PD-1. Given that PD-1 has a cytoplasmic tail (Supplementary Fig. 3) and that c-Cbl is a cytoplasmic protein, we posited that their interaction occurs through the cytoplasmic tail of PD-1. This contention was examined using GST-pull down assays by treating purified recombinant GST-tagged cytoplasmic tail of PD-1 with lysates from RAW264.7 macrophages. The cytoplasmic tail of PD-1 interacted with c-Cbl (Fig. 5C). Similarly, of recombinant GST C- and N-termini of c-Cbl^11^, interaction of PD-1 was observed specifically with the C-terminus of c-Cbl (Fig. 5D). Next, we examined if these two proteins co-localized in BMDM from c-Cbl^**+/+**^ mice. Endogenous PD-1 and c-Cbl showed co-localization within the cytosol (Fig. 5E). Taken together, these results support an interaction of the C-terminus of c-Cbl with the cytoplasmic tail of PD-1.

**Figure 6:**
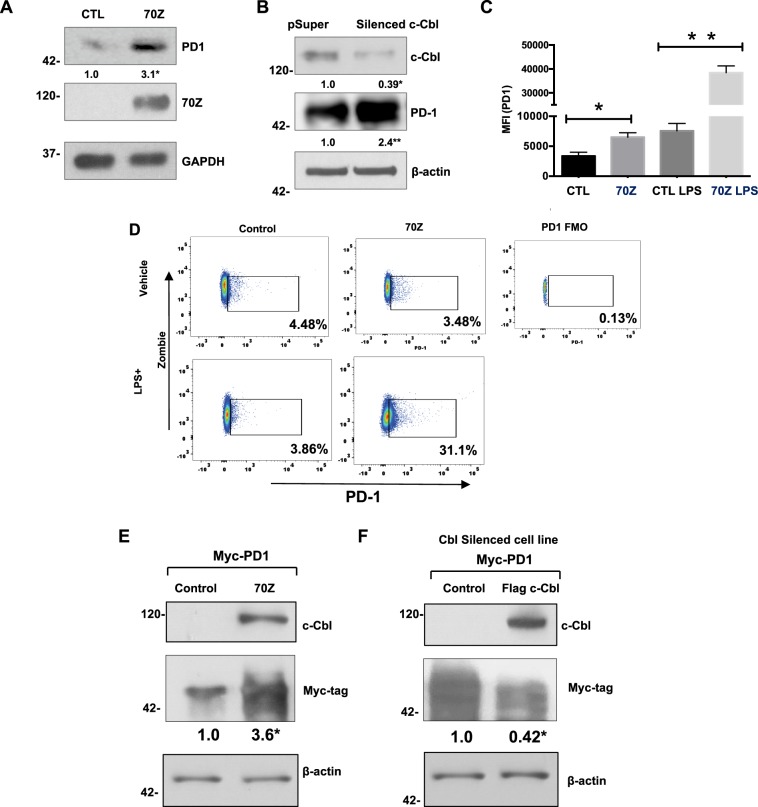
c-Cbl suppresses PD-1. (**A**) RAW264.7 cells transfected with Myc-tagged 70Z-c-Cbl and control (CTL) plasmids were probed separately. Representative immunoblots of five experiments are shown. Student’s t-test was performed. *p = 0.003. (**B**) RAW267.4 cells expressing control (pSuper) or *c*-*Cbl* shRNA (*silenced c*-*Cbl*) were probed. Equal amounts of lysates were probed separately for PD-1. Representative immunoblots from three independent experiments. Student’s t-test was performed. * and **p = 0.001. (**C**) RAW267.4 cells transfected with 70Z-c-Cbl and control plasmids were treated for 16 hours with LPS (1 ug/ml) followed by FACS analysis. The live cells using Zombie were gated for PD-1. MFI of PD-1+ cells from three independent experiments is shown. ANOVA and Student’s t-test were performed. Error bars = SD. *p = 0.03 and **p = 0.001. (**D**) RAW267.4 expressing 70Z-c-Cbl and control plasmids were treated overnight with or without LPS (1 ug/ml) for 16 hours followed by FACS analysis. The cells were gated using Zombie dye and for PD-1. Gates set with PD-1 Fluorescence Minus One (FMO) control. Representative FACS plots from three independent experiments are shown. (**E**) HEK 293 T cells co-expressing Myc-tagged PD-1 and different c-Cbl constructs were lyzed and probed for c-Cbl and actin. Equal amounts of lysates were probed for Myc-tagged PD-1 given close proximity to Actin. A representative of three experiments is shown. Increase in PD-1 was compared using Student’s t-test. *p = 0.01. (**F**) HEK2913T cells silenced for c-Cbl were transfected with Myc-PD-1 and c-Cbl or control vector. The lysates were probed for c-Cbl and Actin. Equal amounts of lysates were probed for Myc-tagged PD-1 given close proximity to Actin. A representative of three experiments is shown. Suppression in PD-1 expression was compared using Student’s t-test. *p = 0.02.

Based on the results of binding assays and that the knowledge that c-Cbl^+/−^ macrophages had increased PD-1, we hypothesized that c-Cbl might suppress PD-1. Reduction in c-Cbl activity with 70Z-c-Cbl or c-Cbl silencing (to an extent similar to c-Cbl^+/−^ immune cells in Fig. 1A) in RAW264.7 significantly upregulated (2-3-fold) endogenous PD-1 (Fig. 6A,B). This effect is demonstrated by the ability of 70Z to bind to but fail to suppress PD-1 (Fig. 5A). Thus, counteracting the regulation of endogenous c-Cbl on PD-1 in macrophages. These data generated in unstimulated macrophages are also consistent with the elevation of PD-1 levels observed in BMDM from uninjected mice (Fig. 4A,C).

We further examined if this increase in PD-1 in whole cell lysate corroborated with the increased surface expression of PD-1. FACS analysis of 70Z-c-Cbl expressing RAW264.7 cells showed doubling of MFI of PD-1 (mean + SEM 7567 + 1247) compared to control (3367 + 636, p = 0.033) (Fig. 6C). Interestingly, this increase was not associated with an augmented percentage of PD-1+ macrophages (Control: 3.79 + 0.38%, 70Z-c-Cbl: 3.15 + 0.20%, p = 0.255) (Fig. 6D). These results suggest that the surface expression of PD-1 was increased in cells transfected with 70Z-c-Cbl. LPS treatment increased both MFI of PD-1 (4–5-fold) and % of PD-1+ cells (7–8-fold). With LPS treatment, a significant increase in MFI of PD-1 was also noted in 70Z-c-Cbl compared to control cells (Control: 7567 + 1247, 70Z-c-Cbl 38433 + 2824, p = 0.001) and increase in % of PD-1+ cells (Control: 4.81 + 0.51%, 70Z-c-Cbl: 31.17 + 3.46%, p = 0.005).

A similar regulation of PD-1 was noted in other cell types. Co-expression of PD-1 and 70Z-c-Cbl in HEK-293T cells increased PD-1 by 3-fold (Fig. 6E). A rescue experiment was performed in *c*-*Cbl* silenced cells transiently co-expressing c-Cbl and PD-1. Expression of c-Cbl restored the suppression of PD-1 (Fig. 6F).

**Figure 7:**
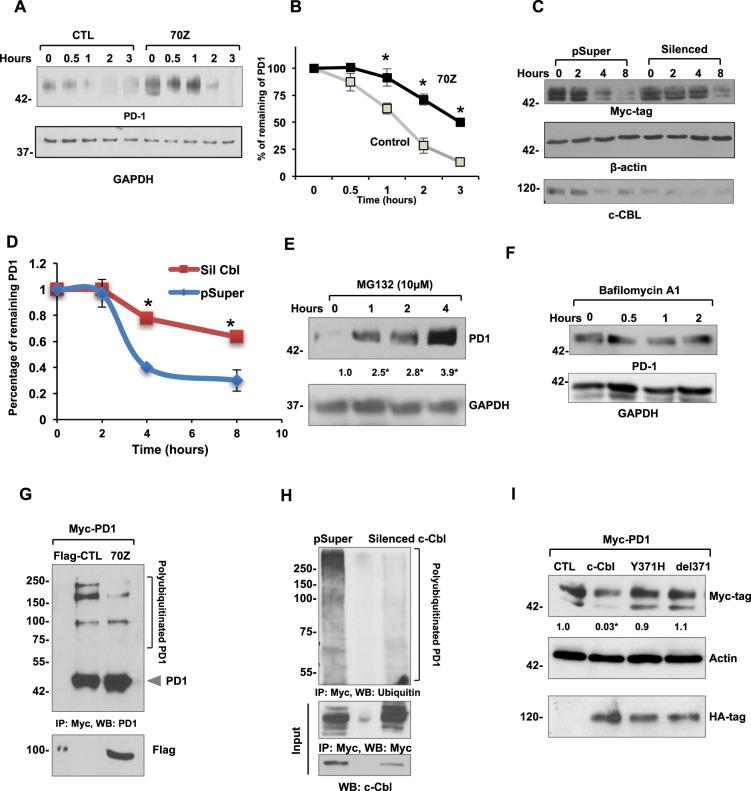
c-Cbl destabilizes PD-1 and targets it for ubiquitination and proteasomal degradation. (**A**) RAW264.7 expressing control (CTL) and 70Z-c-Cbl were treated with Cycloheximide (300 μM) for the indicated time. Representative immunoblots from four experiments are shown. (**B**) Densitometry of PD-1 normalized to GAPDH and represented as the percentage of PD-1 at time = 0. Time to reach 50% of PD-1 was considered as its half-life. Average of four experiments is shown. Error bars = SEM. Student’s t-test was used. *p = 0.03 and **p = 0.01. (**C**) Lysates from HEK-293T cells stably expressing control (pSuper) and c-Cbl shRNA (Silenced) vectors were transfected with Myc-PD-1 and treated with Cycloheximide (100 μM) for the indicated time. The amount of remaining PD-1 was detected by Western blotting. Equal amounts of lysates were probed separately to confirm c-Cbl silencing. A representative figure from four independent experiments is shown. (**D**) Densitometry of normalized PD-1 bands performed using ImageJ represented as the percentage of PD-1 at time 0 is shown. The time to reach 50% of initial PD-1 was considered as the half-life of PD-1. An average of four experiments is shown. Error bars = SD. Student’s t-test was performed. *p = 0.001 and **p = 0.003. (**E**) RAW264.7 cells treated with MG132 for indicated amount of time. Representative immunoblots from four independent experiments are shown. Student’s t-test with Bonferroni correction was used. Compared to time = 0, *p = 0.04 at 1 hour, p = 0.001 at 2 and 4 hours. (**F**) Raw264.7 cells treated with 200 nM of Baflomycin A1 for the indicated time points. The lysates were probed as shown. A representative of two experiments is shown. (**G**) HEK 293T cells expressing control (Flag-CTL) and 70Z-c-Cbl were pre-treated with 10 µM MG132 overnight and immunoprecipitated using PD-1 antibody. The eluents were probed with PD-1. 5% of lysates were probed separately with Flag-tag. Representative immunoblots from three experiments are shown. (**H**) HEK293T cells stably expressing shRNA of c-Cbl (Silenced c-Cbl) and control (pSuper) and transfected with Myc-tagged PD-1 were immunoprecipitated using Myc antibody. Eluents were probed with anti-ubiquitin antibody. 5% of lysates were probed for c-Cbl. Representative immunoblots from three experiments are shown. (**I**) HEK293T co-expressing Myc-tagged PD-1 and HA-tagged c-Cbl or control (CTL) constructs were probed and normalized to actin. Equal amounts of lysates were probed for HA-tag. Representative immunoblots from three independent experiments. 2-way ANOVA with multiple comparison and Student’s t-test were performed. Compared to CTL, *p = 0.02 for c-Cbl.

While no increase in PDL-1 was observed in c-Cbl^+/−^ BMDM (Fig. 4D,E), the levels of PDL-2 were low in c-Cbl^**+/+**^ and c-Cbl^+/−^ BMDMs. In order to examine the effect of c-Cbl on both the ligands of PD-1, we explored 70Z-expressing RAW264.7 cells. Control or 70Z expressing RAW264.7 cells with or without LPS showed no difference in PDL-1 (Supplementary Fig. 3A) or PDL-2 levels (Supplementary Fig. 3B). Taken together, these results suggested that c-Cbl regulates the surface expression of PD-1 but not of its ligands and that PD-1 increase was amplified with pro-inflammatory conditions.

c-Cbl degrades (destabilizes) its target proteins, and this process is measured by half-life studies^11,22^. We examined PD-1 protein half-life in RAW264.7 cells expressing 70Z-c-Cbl. Using Cycloheximide, a protein translational inhibitor, we observed half-life of 72 minutes of endogenous PD-1, which was significantly prolonged by 70Z-c-Cbl to 180 minutes (Fig. 7A,B). Similar results were noted in HEK-293T cells silenced for *c*-*Cbl*, which showed a significant increase in the half-life of overexpressed PD-1 from 3.25 hours to 8 hours (p = 0.02) (Fig. 7C,D). c-Cbl targets proteins for proteasomal or lysosomal degradation^4,11^. We examined both these types of degradation in macrophages using inhibitors. Treatment of RAW264.7 with MG132, a proteasome inhibitor increased PD-1 up to 3-fold within one hour (Fig. 7E). This result corroborates with the half-life of PD-1 (~ 45 minutes) in macrophages (Fig. 7B). No changes were noted with lysosomal inhibitor Bafilomycin A1 (Fig. 7F).

Because c-Cbl is a E3 ubiquitin ligase, we examined the possibility that c-Cbl mediated the ubiquitination of PD-1. This was addressed using both 70Z-c-Cbl and silencing *CBL* and by treating cells with MG-132, a commonly employed agent to block the proteasomal degradation and to increase the ubiquitination signal. Treatment with MG132 showed higher molecular weight bands up to 250  KD of PD-1, suggesting polyubiquitination of PD-1 (Fig. 7G,H). This signal was reduced by 72–80% (p = 0.008) with 70Z-c-Cbl. Upon probing with a ubiquitin antibody, PD-1 polyubiquitination appeared as a smear, which was also reduced by 90% with *c*-*Cbl* silencing (p < 0.001) (Fig. 7H). These experiments suggest that c-Cbl destabilizes PD-1 and targets it for ubiquitination and proteasomal degradation.

We further probed if c-Cbl-mediated PD-1 suppression was dependent on c-Cbl’s E3 ligase activity. A naturally-occurring oncogenic point mutant of c-Cbl c.1111 T > C (p.371Y > H) with compromised ubiquitin E3 ligase activity was used^12^. X-ray crystallography studies have shown that this alteration imparts an auto-inhibited conformation to c-Cbl suppressing its ubiquitin ligase activity^23^. Our results showed that unlike wild-type c-Cbl, the cells expressing c-Cbl Y371H or del371 failed to downregulate PD-1 (Fig. 7I). Consistent with the binding and lack of activity of 70Z-c-Cbl on PD-1 (Fig. 6A,C), these results underscore the need for an intact RING finger domain activity of c-Cbl to suppress PD-1.

## Discussion

**Figure 8:**
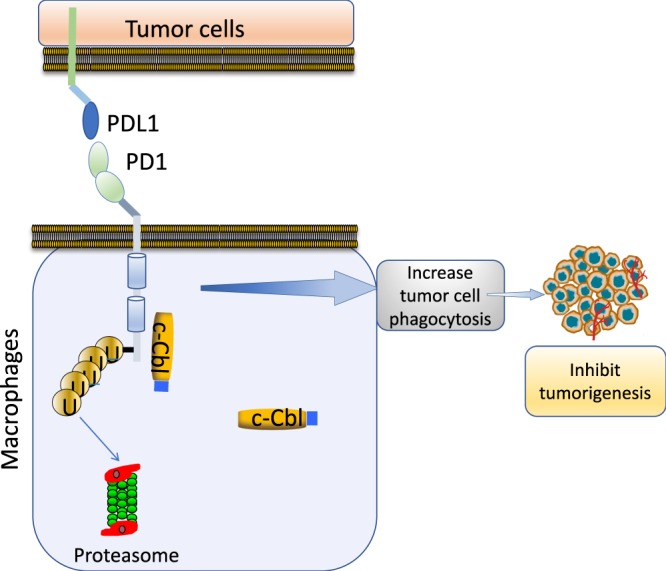
Model of c-Cbl mediated PD-1 regulation. c-Cbl in immune cells (such as macrophages) binds and ubiquitinates PD-1 to target it for proteasomal degradation. This results in downregulation of surface expression of PD-1, which in macrophages augments the tumor cell phagocytosis resulting in suppression of tumor growth.

This study advances our understanding on the role of c-Cbl in tumorigenesis on several fronts. Previous work has shown that c-Cbl exerts its tumor suppressive effect by downregulating oncoproteins such as Wnt/β-catenin as well as receptor- and non-receptor tyrosine kinases in cancer cells^4,7,9,24^. c-Cbl has also been shown to impede tumor-associated angiogenesis^7,8^. The current work adds a previously unrecognized immunologic component to the tumor suppressive effect of c-Cbl. c-Cbl binds to the intracytoplasmic tail of PD-1 and targets it for ubiquitination-proteasomal degradation in macrophages, resulting in downregulation of PD-1 and reduced surface expression leading to increased tumor phagocytosis and tumor suppression (Fig. 8). While c-Cbl is a well-established signaling molecule in the context of central tolerance in the thymus^5^, we demonstrate a role of c-Cbl in the periphery such as in TAMs, a cell type which is increasingly targeted for therapeutic manipulation^25^.

This study also provides new insights into biology of PD-1 and identifies c-Cbl as a novel ubiquitin E3 ligase of PD-1. The C-terminus of c-Cbl contains several domains including a proline-rich region, which is known to interact with SH3-containing proteins^4^. It also has three tyrosine residues, which upon phosphorylation binds to SH2 domain-containing signaling molecules^4^. Moreover, PD1 is known to be phosphorylated at the tyrosine site^13,26^. The interaction of c-Cbl and PD-1 was observed using recombinant protein of C-terminus of PD1 generated in *E*. *coli*, which is likely not phosphorylated. These observations suggest that the c-Cbl-PD-1 interaction may not depend on the phosphorylation status of PD-1 and may point to the interaction of PD-1 on the proline rich region of c-Cbl.

A recent study demonstrated that PD-1 undergoes ubiquitination by an E3 ubiquitin FBXO38, a multiprotein complex belonging to the SKP1–CUL1–F-box protein (SCF) family of ligases^27^. In activated T-lymphocytes, surface PD-1 undergoes internalization, subsequent ubiquitination, and proteasome degradation by FBXO38. Having more than one E3 ubiquitin ligase is not surprising. Important signaling proteins are known to be targeted by a set of E3 ubiquitin ligases that differ in terms of their binding to the target protein and degrade the target in a cell-type specific manner or in the context of upstream signaling. For example, oncogene β-catenin can be ubiquitinated by various E3 ubiquitin ligases namely β-TrCP, Jade-1, Ozz, and c-Cbl. They have distinct roles in regulating different species of β-catenin in different subcellular compartments depending on the phosphorylation status of the N-terminus of β-catenin^11,22,28^. More studies are needed to compare the relative roles of these two E3 ubiquitin ligases of PD-1 in immune cells and whether their interaction or subsequent degradation of PD-1 differs based on PTMs of PD-1 or signaling events. It is interesting to note that CBL families of protein also regulates the PDL-1/PDL-2 in different cell types. For example, in wild-type EGFR lung cancer cell lines A549 and H460, both Cbl-b and c-Cbl inhibited PD-L1 by inactivating STAT, AKT, and ERK signaling^20^. Along with these previous studies, the current manuscript demonstrates a direct role of c-Cbl in PD-1 degradation and raises a possibility of CBL family of proteins in regulation of other immune checkpoints.

c-Cbl is likely to suppress PD-1 in different states of immune cells. For example, PD-1 expression is maintained at low levels in unstimulated macrophages and is induced in the immune cells of the inflammatory milieu and tumor microenvironment. It is conceivable that in unstimulated macrophages, c-Cbl binds to the cytosolic tail of PD-1 and maintains a low level of PD-1, which may represent a physiologic function of c-Cbl in PD-1 regulation. This possibility is supported by regulation of PD-1 by c-Cbl in unstimulated macrophages (Fig. 4A,C). That the effect of c-Cbl on PD-1 is several folds increased following pro-inflammatory cytokine stimulation (Figs. 4C, 6C) supports the notion that c-Cbl downregulates PD-1 in the inflammatory milieu to possibly prevent its uncontrolled expression. More studies are needed to probe these possibilities and other questions such as c-Cbl’s regulation of PD-1 in different subsets of macrophages. This work was focused on macrophages and was motivated by the expression of c-Cbl in myelomonocytic cell lines and its mutation patterns in humans^3,4,12^. However, c-Cbl is also expressed in T-lymphocytes, which are altered in the tumor infiltrates of c-Cbl^+/−^ mice and may contribute to the augmented tumor growth in them (Figs. 2C,D and 3C,D). Further work is needed to probe the effects of c-Cbl perturbation in different cell-types such NK cells or T-lymphocytes using chimeric mice or conditional cell-type specific KO mice.

The membrane proteins undergo either lysosomal or proteasomal degradation and the lysosomes play critical role in endocytosis of integral membrane proteins. The fate of the protein to either type of degradation depends in part by the type of ubiquitination such as polyubiquitination and monoubiquitination and the nature of ubiquitination chain at specific lysines K48 v/s K63 on the ubiquitin moiety^29^. It is known that c-Cbl can target proteins to either types of degradation^30^. Using specific lysosomal inhibitors (Bafilomycin), our data showed that PD-1 is targeted for proteasomal but not the lysosomal degradation (Fig. 7E,F). Future studies will probe the specific signals that target PD-1 to the proteasomal degradation and also clarify questions related to mechanisms of increased immune infiltration in tumors with the reduction in c-Cbl activity, as this can be potentially leveraged to augment the efficacy of anti-PD-PDL-1 therapies.

Despite an unprecedented clinical success, not all CRC patients respond to anti-PD-1-PDL1 therapeutics underscoring a need for other therapeutic targets that perturb several aspects of tumorigenesis. c-Cbl represents such a target with multi-pronged effects on cancer cells, angiogenesis and now immune cells. The current work strongly advocates for further probing of the mechanisms of degradation of other immune checkpoint proteins and their therapeutic relevance; as well as for the investigation of a potentially broader role that c-Cbl may play in other conditions driven by immune checkpoint abnormalities such as autoimmune diseases and viral infections.

## Methods

### Flow cytometry

After washing, tumor cells were re-suspended in PBS (Boston BioProducts, BM-222). Non–specific binding of Fc receptors by immunoglobulin was blocked using anti-CD16/32 (Biolegend, 101302). Live/Dead staining was conducted using Zombie UV Fixable Viability kit (Biolegend, 423107). Cells were washed in PBS, centrifuged at 1500 RPM, and re-suspended in FACS buffer (PBS with 0.5% BSA and 2 mM EDTA). Surface marker cellular staining was conducted using the following fluorescently conjugated antibodies (BioLegend): anti-CD45 (103137), anti-CD3 (100227), anti-CD4 (100405), anti-CD8 (100765), anti-F4/80 (123145), and anti-PD1/ CD279 (135209).

### Isolation of bone marrow derived macrophages (BMDM)

 Mice were sacrificed by carbon dioxide followed by cervical dislocation. Hind legs were removed and quickly submerged in 70% ethanol followed by submersion in ice-cold PBS. Skin was removed and the muscles were stripped from the bones. A 25-guage needle syringe (BD) was used to flush out bone marrow into a sterile Petri dish (35/10 mm) containing 5 ml sterile ice-cold PBS. Cell suspension was collected and centrifuged 7 minutes at 1500 RPM. The supernatant was removed and the pellet was re-suspended in 5 ml ACK Lysing Buffer (Gibco, A10492-01) for 2 minutes followed by addition of 35 ml sterile PBS. Cell suspension solution was filtered through a 70-micron cell strainer (Denville, TC1070-A) and centrifuged for 7 minutes at 1500 RPM. The cell pellet was re-suspended in 4 ml BMDM media (complete DMEM (Gibco. 1195-040) with L929 derived M-CSF). 1 ml of cell suspension was added to 30 ml of BMDM media in 150 mm untreated cell culture plates (Denville, 229655). Cells were incubated for 3 days at 37 °C, 5% CO_2_. On the third day, 10 ml of fresh BMDM media was added to the cells and incubation was continued for an additional four days. Adherent macrophages were observed by day 3 with complete plate confluency by day 7.

Detailed methods, including the animal experiments, phagocytosis assay, immunoblotting, immunoprecipitation and ubiquitination and other methods relevant to this paper, including statistical analysis are in the Supplementary Information. All animal experiments were carried out in accordance with relevant guidelines and regulations. All the protocols were approved by the Institutional Animal Care and Use Committee of Boston University Medical Center (AN-15497).

## Supplementary information

Supplementary information 
