# Supplementary material for: c-Cbl targets PD-1 in immune cells for proteasomal degradation and modulates colorectal tumor growth
Source: Sci Rep. 2019 Dec 27;9:20257. doi: 10.1038/s41598-019-56208-1 (PMC6934810; doi:10.1038/s41598-019-56208-1)

c-Cbl targets PD-1 in immune cells for proteasomal degradation and modulates colorectal tumor growth

Chimera Lyle[1]*, Sean Richards[1]*, Kei Yasuda[1], Marc Arthur Napoleon[1], Joshua Walker[1], Nkiruka Arinze[2], Mostafa Belghasem[3], Irva Vellard[1], Wenqing Yin[1], Jonathan D. Ravid[4], Elias Zavaro[1], Razie Amraei[3], Jean Francis[1], Uma Phatak[2], Ian R. Rifkin[1,5], Nader Rahimi[2], Vipul C. Chitalia[1,5,6]

*These authors equally contributed

Supplementary methods section

Generation of c-Cbl$^{+/-}$ mice

All the mice used in this study were bred and maintained at Boston University Medical Center after approval from Institutional Animal Care and Use Committee (AN-15449). c-Cbl$^{+/-}$ male and female mice on C57/BL6J background were obtained from Dr. Chiang Jeffery, National Cancer Institute. Due to ectoparasite infection in these animals, according to the policy of Institutional Animal care and Use committee of Boston University School of Medicine, a clean line of c-Cbl$^{+/-}$ mice was re-derived using standard IVF protocol. Briefly, male mice euthanized and sperm were extracted and preincubated in a medium with 0.25 mM GSH (glutathione)/Cook medium. After confirming the sperm recovery based on sperm 100% motility, the IVF was performed. Donor eggs were obtained from C57BL/6 female mice (Jackson lab catalogue 000664 B6/J), which is the same background as original c-Cbl$^{-/-}$ mice. Fifteen 5-week old females with moderately expanded or nearly unexpanded ampulla were used and 235 oocytes/15 donors. A total of 156 zygotes were confirmed using polar bodies with 66% fertilization rate. All the embryos were distributed evenly among 5 plugged recipient mothers with nearly 16 embryos per oviduct.

Subsequent offspring were genotyped using a pair of primers for the wild type allele

CBL wt fwd: 5’-GACGATAGTCCCGTGGAAGAGCTTTCGACA-3’

CBL wt rvs: 5’-CCTAAGTGGTAGGATTATAATTGCAAGCCACCA-3’),

and a set of primers for the knockout allele

CBL KO fwd: 5’-TCCCCTCCCCTTCCCATGTTTTTAATAGACTC-3’

and LOX-P: 5’-TGGCTGGACGTAAACTCCTCTTCAGAACCTAATAAC-3’)

Xenograft Animal Model

MC-38 murine colon adenocarcinoma cells derived from C57BL6 mice were cultured in vitro in 10% FBS (Denville, FB5001), 1% L-Glutamine (Corning, 25-005-CI), and 1% Penicillin-Streptomycin (Corning, 30-002-CI) supplemented “complete” RPMI (HyClone, SH30255.01). c-Cbl +/+ and c-Cbl +/- mice were injected subcutaneously with $0.5x10^6$ tumor cells. Tumors were allowed to develop and measurements (length x width) were taken at intervals of 3 days using a caliper. The volume of tumor was calculated using the formula ½ x D x d^2 where *D* is the major axis of the tumor and *d* is the minor axis. Mice were sacrificed at the end of 3 weeks or when ulceration of the tumor occurred. Following harvest, tumors were manually dissected and minced before digestion with collagenase type IV (270 U/mg, Worthington Biochemical Corporation, LS004186) for 30 minutes at 37°C37˚C humidified incubators in the presence of 5% CO_2. The digested tumors were processed to encourage an easy flowing single cell suspension and then filtered through a 70-micron filter (Denville, TC1070-A) followed by centrifugation for 10 minutes at 1200 RPM and washing/resuspension of the pellet in 8ml buffer (PBS with 5% FBS).

Cell culture

RAW 264.7 cells were obtained from ATCC (# TIB-71) and cultured in DMEM media (ATCC-30-2002) containing reduced level of sodium bicarbonate formulated for use in 5% CO_2 incubators. Cells were cultured in 150mmx 20mm treated tissue culture dishes (Denville, 170704-116A) in complete DMEM in 37˚C humidified incubators in the presence of 5% CO_2. Before conducting experiments, 1 mL of cells (density 3 X 10^6 cells/mL) were seeded in 6 well culture plates (Denville, T1006) containing 4 ml of complete DMEM. Cells were allowed to rest and adhere for 2 hours. LPS from *Escherichia coli* 0114: B4 (Sigma, L2630) was dissolved in PBS to a concentration of 100ng /uL or 1ug/uL. MC38 cell line obtained from ATCC was grown in RPMI

medium supplemented with 10% FBS and 1% Penicillin and streptomycin. The cells were treated with MG132 (CalBiochem) and BafilomycinA1 (Tocris Bioscience CAS 88899-55-2) dissolved in DMSO.

Phagocytosis assay using confocal microscopy BMDMs were harvested from the femurs of mice. Myeloid cells were differentiated as above for seven days. On the seventh day, $2x10^6$ cells were harvested and plated on sterile glass cover slips (Fisher Scientific, 12-548 B) in sterile 6 well cell culture plates (Thomas Scientific, 1156D98) in complete DMEM medium. Cells were allowed to adhere overnight. Plated BMDMs were treated overnight with 8.5 ug anti PD-1 blocking antibody (BioXcell, BE0273) or 8.5 ug anti Rat IgG2a isotype control (BioXcell, BE0089). Plates were gently washed with sterile room temperature PBS and fresh complete DMEM was added. Cells were then co-cultured 3 hours with mouse normal serum opsonized GFP-MC38 ($1.5x10^6$) adenocarcinoma cells. After 3hours, media was removed from wells and cover slips containing adherent cells were gently washed twice in warm PBS.

Cells were incubated for 10 minutes in PBS containing 0.3% Triton X-100 (American Bioanalytical, 9002-93-1). Cells were then washed three times for 5 minutes in PBS. After washing, cells were blocked in 1% BSA in PBST containing CD16/32 (10ug/ml) for 30 minutes. Cells were briefly washed once in PBS. APC anti-mouse CD11(Biolegend, 101211) was diluted in 1% BSA in PBST to a concentration of 2.5ug/ml and 1 ml was added to each well containing a cell adhered coverslip. Plates were incubated at room temperature in the dark for 30 minutes, rinsed twice in warm PBS, and covered in aluminum foil for confocal imaging using Leica SP5 microscope.

Immunoblotting and immunoprecipitation were performed as described previously (1-4). All the antibodies for immunoblot and immunoprecipitation were acquired from Cell Signaling and used at the recommended concentrations. The immunoblots were developed using X-ray films or were captured using the BIORAD gel imaging system.

Immunofluorescence and Immunohistochemistry and quantification: IF and IHC was performed on paraffin embedded or fixed cells in paraformaldehyde, as described previously (1-4). For IF on the xenograft tumors, we used CD45 (Cell signaling catalogue number 70257), CD3 (BD Pharmingen, catalogue number 550275) and CD163 (Invitrogen, catalogue number 14-1631-82) antibodies at 1:100 dilution. Alexa fluro AF488 and AF594 secondary antibodies were used and DAPI was used for staining the nuclei.

For image quantification, the entire slide of the tumor xenografts was scanned by a motorized stage system using the Nikon NIS Elements software at the Boston University School of Medicine Imaging core. The images were processed in ImageJ where the signal was converted to gray scale and thresholding was performed equally for all the images. The number and intensity of pixel were analyzed as integrated density. The tumor area was calculated by manually defining the tumor edges. The area of region of interest (ROI) was measured using ImageJ. The integrated density of all the images were normalized to its area.

Ubiquitination assay

Ubiquitination assay was performed as described previously. The cells were treated with MG132 (CelBiochem) 10 uM for indicated time and the harvested cells were subjected to immunoprecipitation. The eluent resolved on SDS page gel was probed for anti-ubiquitin antibody.

GST purification and binding assay

Purification of recombinant GST-tagged protein was performed as described previously (1, 2). Briefly, the C-terminus tail of PD-1 was cloned into pGEX-2 vector using following primers.

Forward primer: TA AGA ATT CGC TCC CGG GCC GCA CGA GGG ACA

Reverse complementary primers:

AGTTTAAA-GCGGCCGC-GAG-GGGCCAAGAACGGTGTCCATCCTC

The product and the vector were digested using ECOR-1 and NOT-1 restriction enzymes (New England Biolab) using manufacturers instruction. The protein synthesis was induced using IPTG and the GST-beads (Amersham) were used to purify the recombinant protein. For in vitro binding assay, GST-tagged protein beads were incubated with cell lysate at 4^0c overnight. The beads were washed using RIPA buffer and eluents were probed for the binding protein.

Constructs

A human PD-1 construct in pCMV3 vector with C terminus Myc-tag was obtained from Sino Biologics. All c-Cbl constructs are previously described (1, 2).

RT-PCR

RNeasy mini kit (Qiagen) was used to extract total RNA from the spleen. 0.5 ug of total RNA were converted to cDNA using Sensiscript RT kit (Qiagen) followed by RT-PCR using pre-validated mouse c-Cbl (Applied Biosystems). *GAPDH* served as a loading control. Levels of mRNA of c-*CBL* were determined using comparative Ct method.

Organs of c-Cbl$^{+/+}$ and c-Cbl$^{+/-}$ were harvested and stored at -80 degrees until RNA extraction was performed. RNA was extracted using the RNeasy Mini Kit from Qiagen (catalogue # 74104). 15mg of tissue was homogenized in 350uL of RLT buffer, and processed according to

the kit's protocol. Concentration and purity of the RNA was measured, and cDNA generated using reverse transcription PCR kit from Applied Biosystems (catalogue # 4368814). c-Cbl mRNA was quantified by quantitative real time PCR using the animals' cDNA and taq-man gene expression probes from Thermo Fisher (catalogue # 4331182).

Statistical methods

Summary statistics are presented using the mean, median, and standard deviation (SD) and standard error of mean (SEM). Either ANOVA with multiple comparison, Student's t-test or Mann-Whitney U test was performed to compare the groups as appropriate. A p-value of less than 0.05 was considered significant.

Supplementary Figure 1

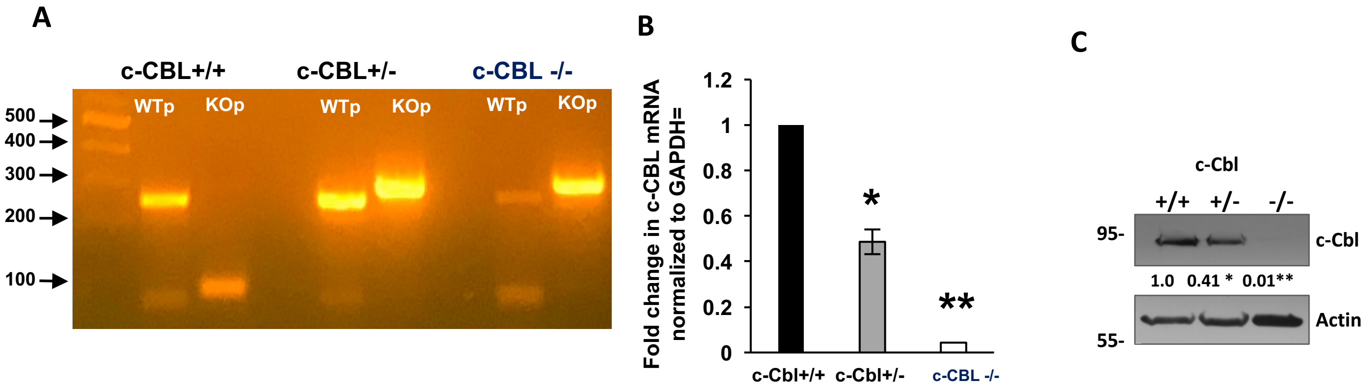

(**A**). Genotyping of animals was performed using their tail clips. Extracted DNA was amplified using the primers for wild-type CBL (WTprimer) and the mutant CBL gene (KOprimer) as described in the method section. A representative DNA gel from c-Cbl$^{+/+}$, c-Cbl$^{+/-}$ and c-Cbl$^{-/-}$ mice is shown.
(**B**). Spleen c-Cbl$^{+/+}$, c-Cbl$^{+/-}$ and c-Cbl$^{-/-}$ mice were harvested and CBL mRNA was quantitated using RT-PCR. GAPDH served as a loading control. Average of three independent experiments is shown. Error bars = SEM. Student's t-test was used. Compared to c-Cbl$^{+/+}$ * $p = 0.002$ and ** $p < 0.001$.
(**C**) Splenic lysates from c-Cbl$^{+/+}$, c-Cbl$^{+/-}$ and c-Cbl$^{-/-}$ mice were probed for c-Cbl. Actin served as loading control and to normalize c-Cbl bands. The normalized c-Cbl values are depicted below the c-Cbl blot. Representative images from three pairs of mice is shown. Student's t-test was used. * and ** $p = 0.001$.

Supplementary Figure 2

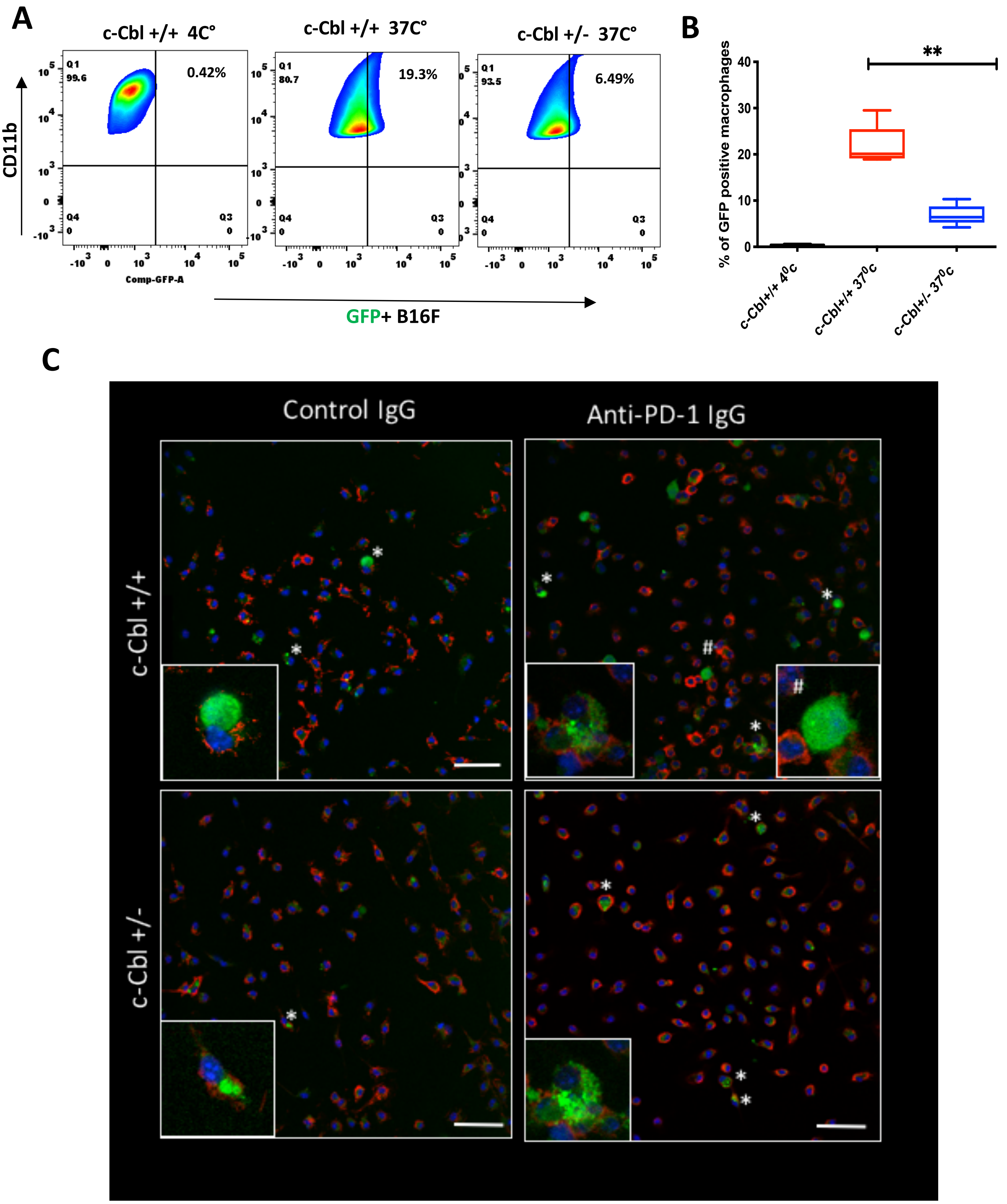

Supplementary Figure 2

D

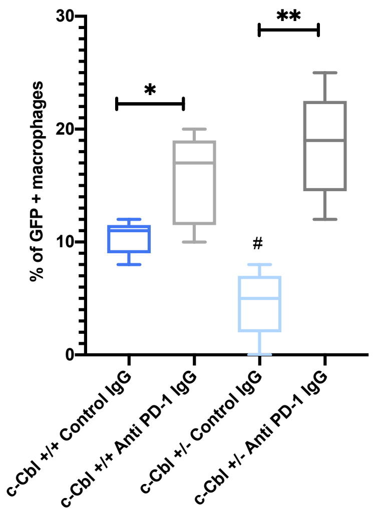

E

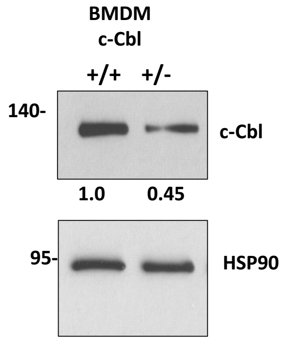

(**A**). BMDM differentiated from c-Cbl$^{+/+}$ and c-Cbl$^{+/-}$ for 7 days were co-cultured with GFP+ B16F tumor cells for 4 hours at indicated temperature. Representative FACS from three independent experiments is shown.

(**B**). BMDM differentiated from c-Cbl$^{+/+}$ and c-Cbl$^{+/-}$ for 7 days were co-cultured with GFP+ MC38 tumor cells for 4 hours and treated with isotype control or anti-PD-1 neutralizing antibodies. Representative FACS from three independent experiments is shown.

(C). Tumor phagocytosis assay. BMDMs obtained from three mice per group were stained for PD-1 and exposed to GFP+ MC38 colon adenocarcinoma cells in the presence of anti- PD1 neutralizing antibody. The isotype antibody served as controls. Representative images from five randomly obtained ones are shown. The white asterisks correspond to PD-1+ GFP+ cells. The insert is a zoomed-in image of a representative macrophage positive for GFP+ MC38 cells. The # symbol corresponds to a non-phagocytosed GFP+ tumor cell, and these were excluded from the analysis. Scale bar = 50 micron.

(**D**) The percentage of GFP+ PD-1+ cells out of PD-1+ cells is shown in a box plot. The horizontal line in the box corresponds to the median, the box spans the 25th and 75th percentile and whiskers represent the maximum and minimal values. ANOVA was applied for the entire group. P = 0.001. Unpaired student t-test was applied to compare the groups.

(**E**). BMDM obtained from two groups of mice were differentiated for 7 days and lyzed. The lysates were probed separately for c-Cbl and HSP90 due to proximity in their molecular weights. Representative images from three mice in each group are shown. The values below the c-Cbl blot represents the densitometry of c-Cbl expression normalized to HSP90.

Supplementary Figure 3

```
        10         20         30         40         50
MQIPQAPWPV VWAVLQLGWR PGWFLDSPDR PWNPPTFSPA LLVVTEGDNA
        60         70         80         90        100
TFTCSFSNTS ESFVLNWYRM SPSNQTDKLA AFPEDRSQPG QDCRFRVTQL
       110        120        130        140        150
PNGRDFHMSV VRARRNDSGT YLCGAISLAP KAQIKESLRA ELRVTERRAE
       160        170        180        190        200
VPTAHPSPSP RPAGQFQTLV VGVVGGLLGS LVLLVWVLAV ICSRAARGTI
       210        220        230        240        250
GARRTGQPLK EDPSAVPVFS VDYGELDFQW REKTPEPPVP CVPEQTEYAT
       260        270        280
IVFPSGMGTS SPARRGSADG PRSAQPLRPE DGHCSWPL
```

Schematic of the PD-1 sequence. PD-1 consists of a single N-terminal immuno-globulin variable region (IgV)–like domain, a transmembrane domain, and a cytoplasmic tail containing tyrosine-based signaling motifs. The cytoplasmic tail contains N-terminal sequence VDYGEL called immunoreceptor tyrosine-based inhibition motif (ITIM), and a C-terminal sequence TEYATI, which forms an immunoreceptor tyrosine-based switch motif (ITSM). The intramembranous part is highlighted with blue color. The intra cytosolic tail has two lysine residues (highlighted in yellow), which are known to be a target of polyubiquitination.

Supplementary Figure 3

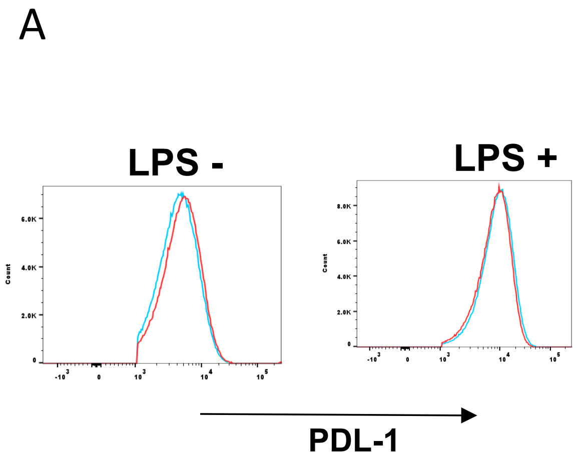

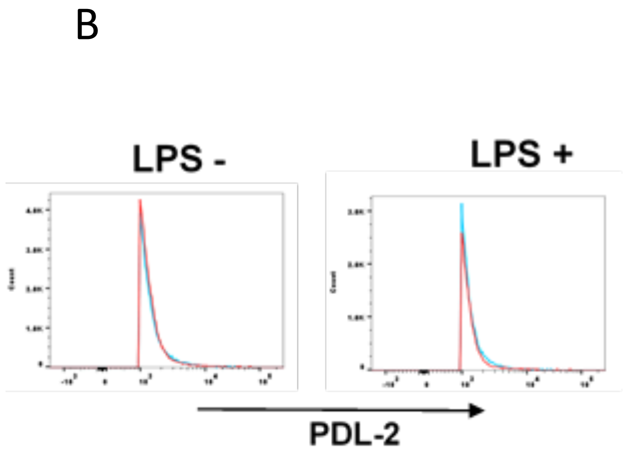

(**A**) RAW267.4 expressing 70Z-c-Cbl (red line) and control plasmids (blue line) were treated overnight with or without LPS (1ug/ml) for 16 hours followed by FACS analysis. Representative MFI of PDL-1 from three independent experiment is shown.

(**B**) RAW267.4 expressing 70Z-c-Cbl (red line) and control plasmids (blue line) were treated overnight with or without LPS (1ug/ml) for 16 hours followed by FACS analysis. Representative MFI of PDL-2 from three independent pairs is shown.

Figure 4C

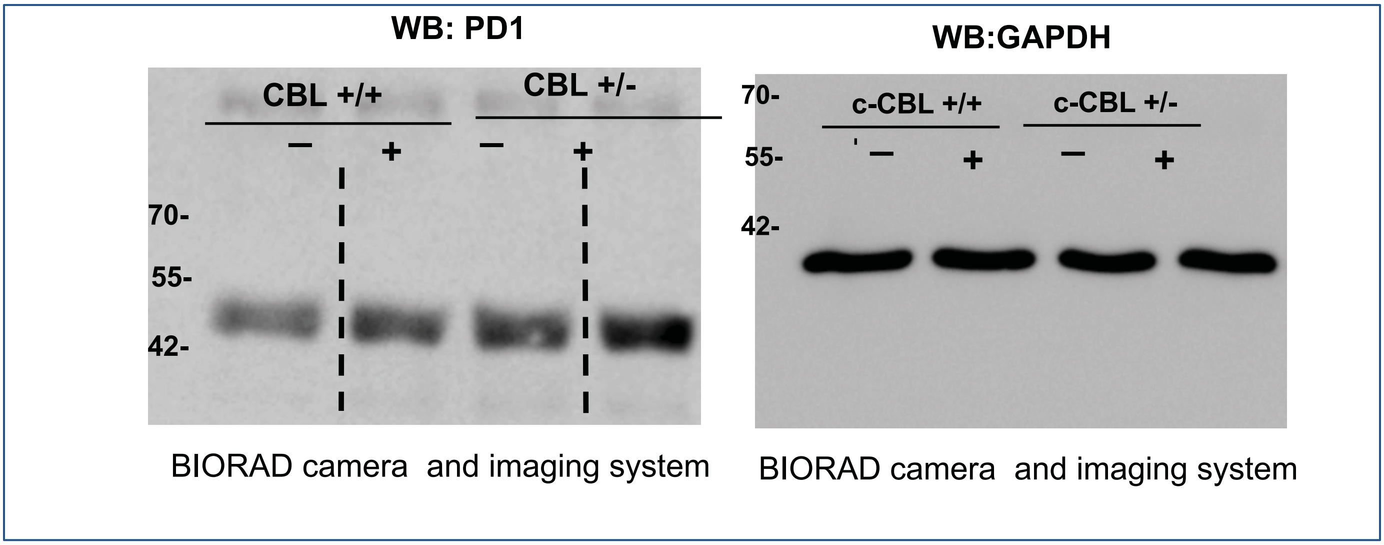

Figure 5A

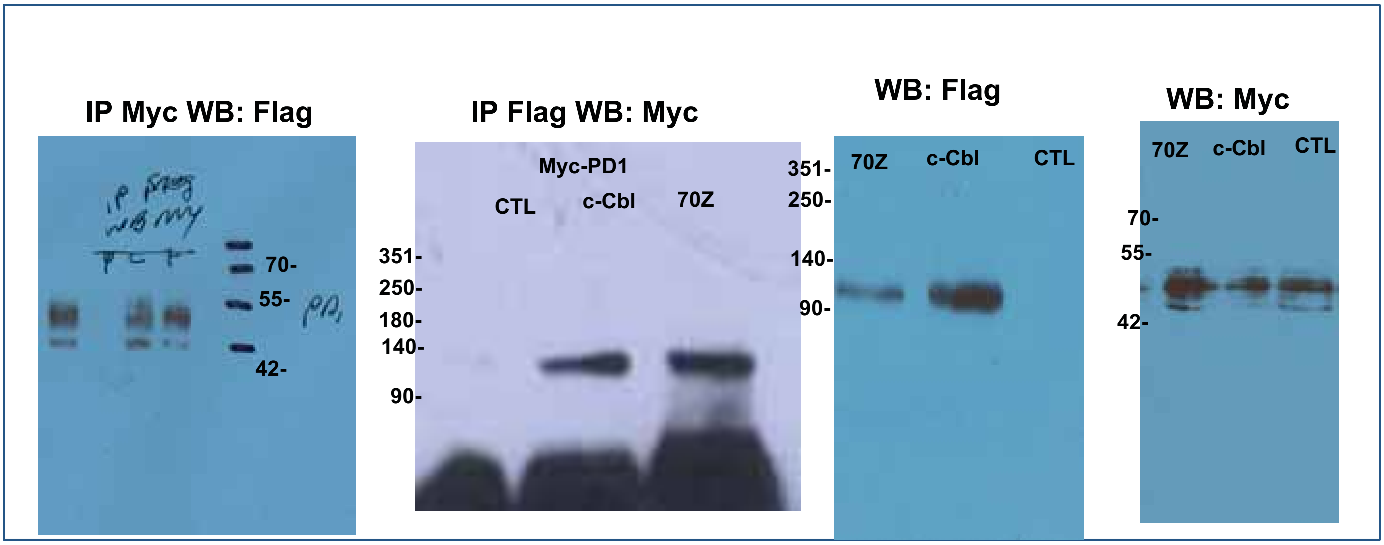

Figure 5B

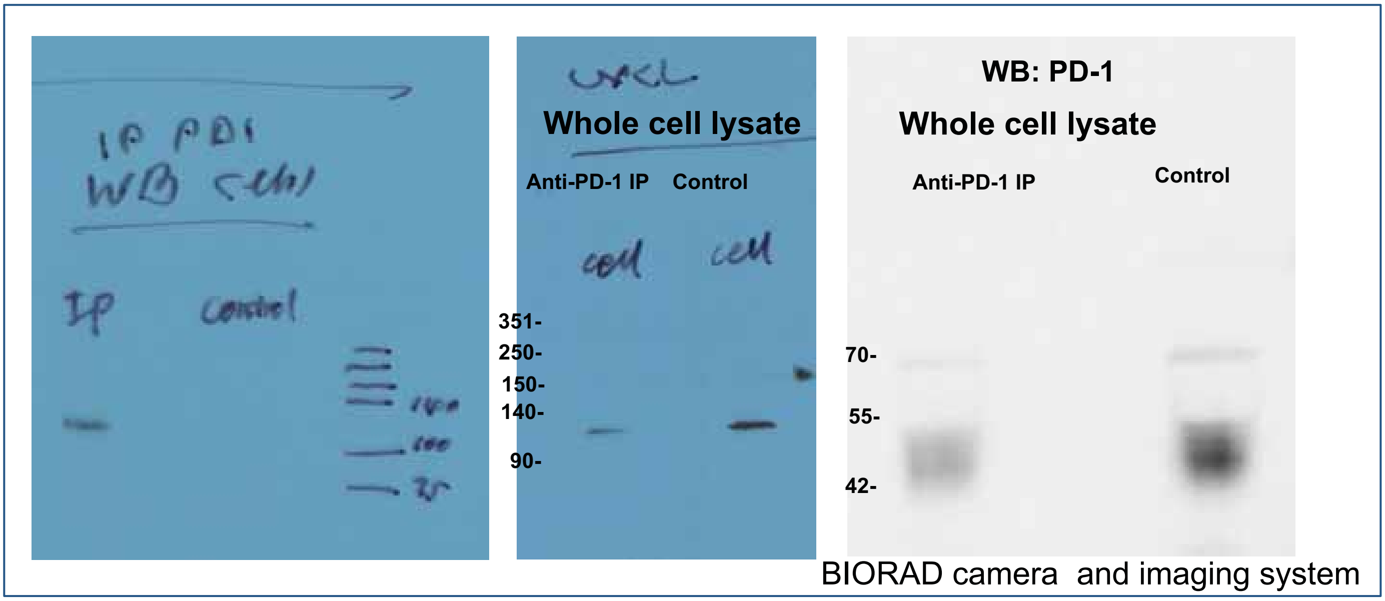

Figure 5C

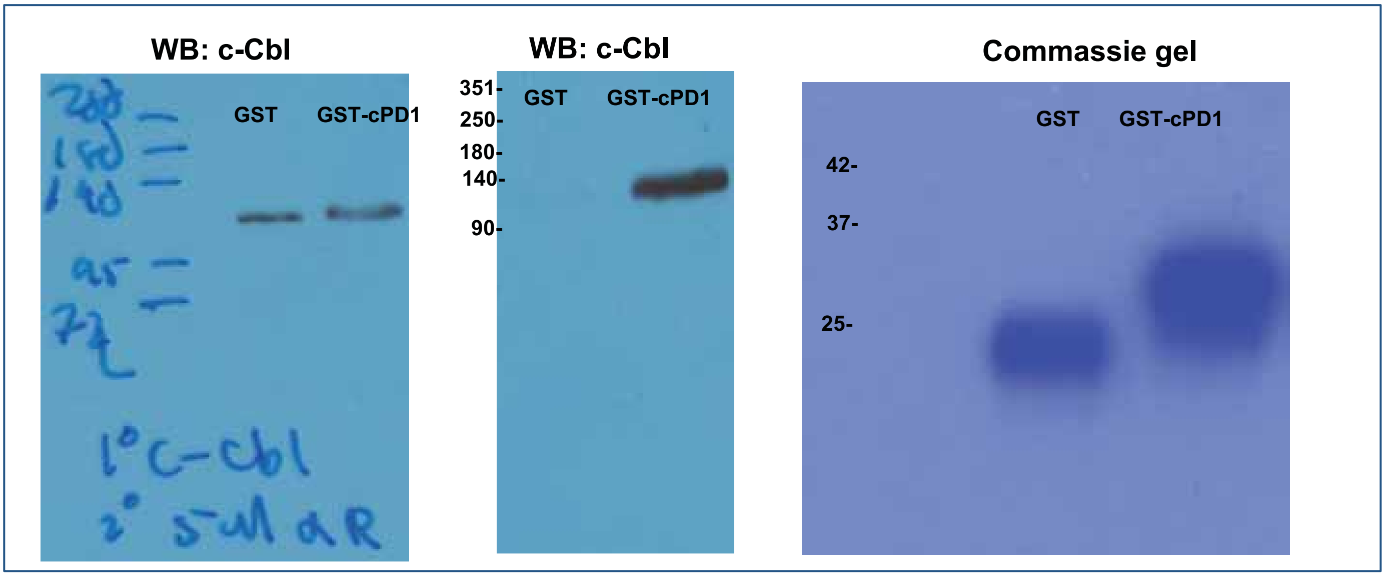

Figure 5D

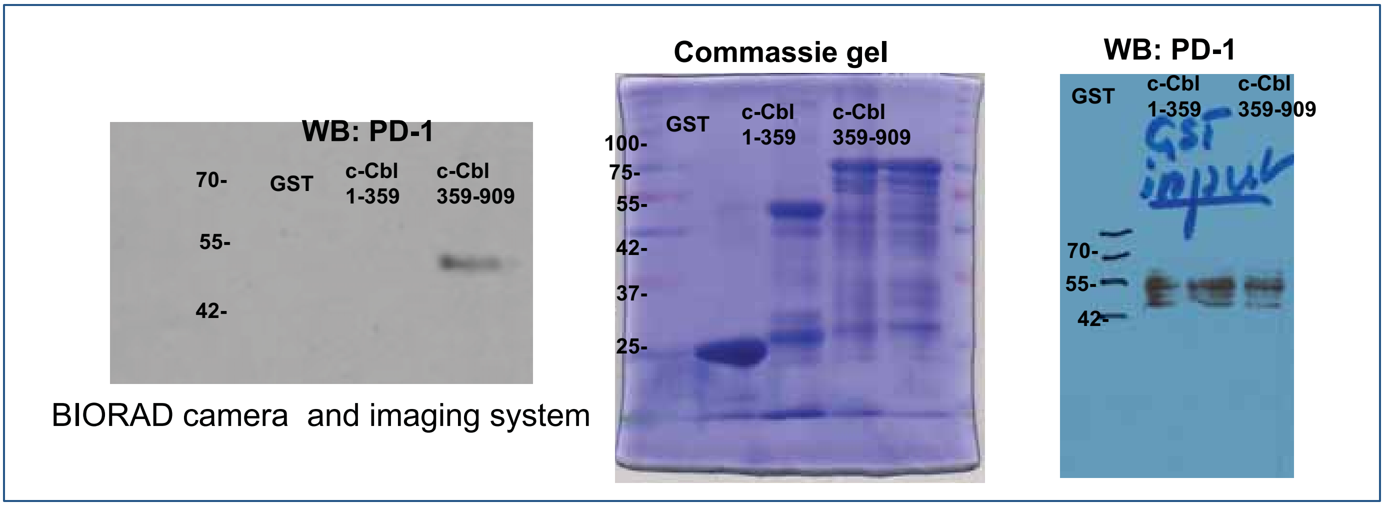

Figure 6A

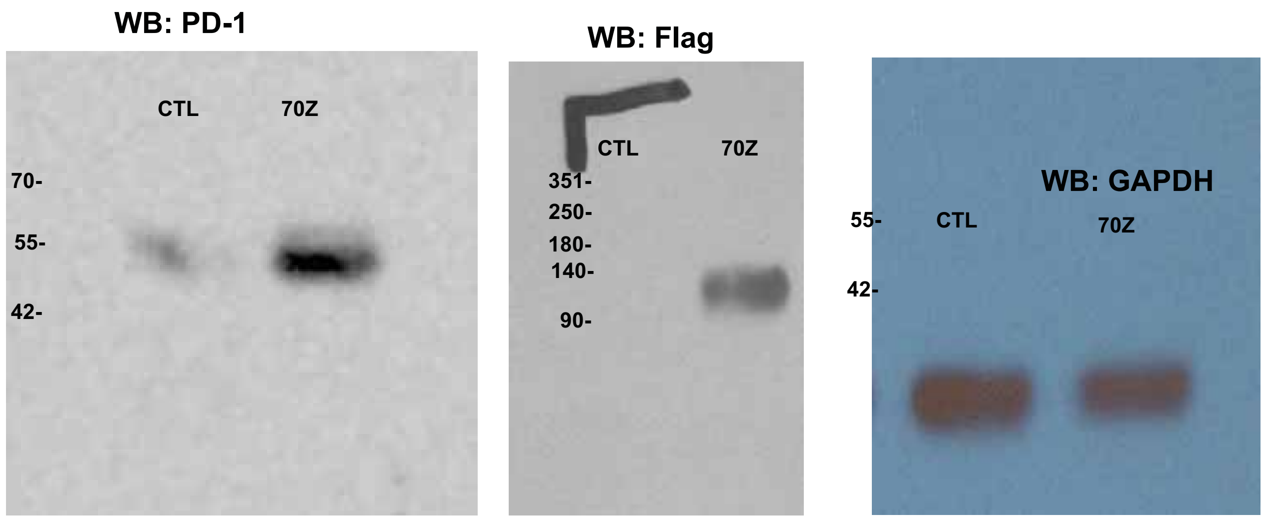

BIORAD camera and imaging system

Figure 6B

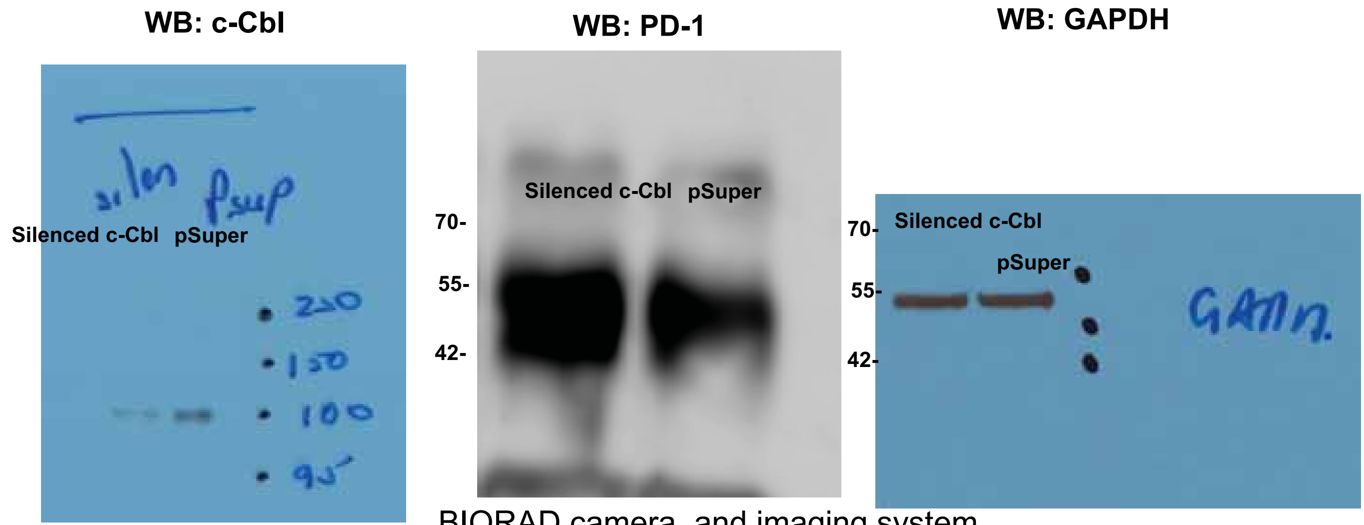

BIORAD camera and imaging system

Figure 6E

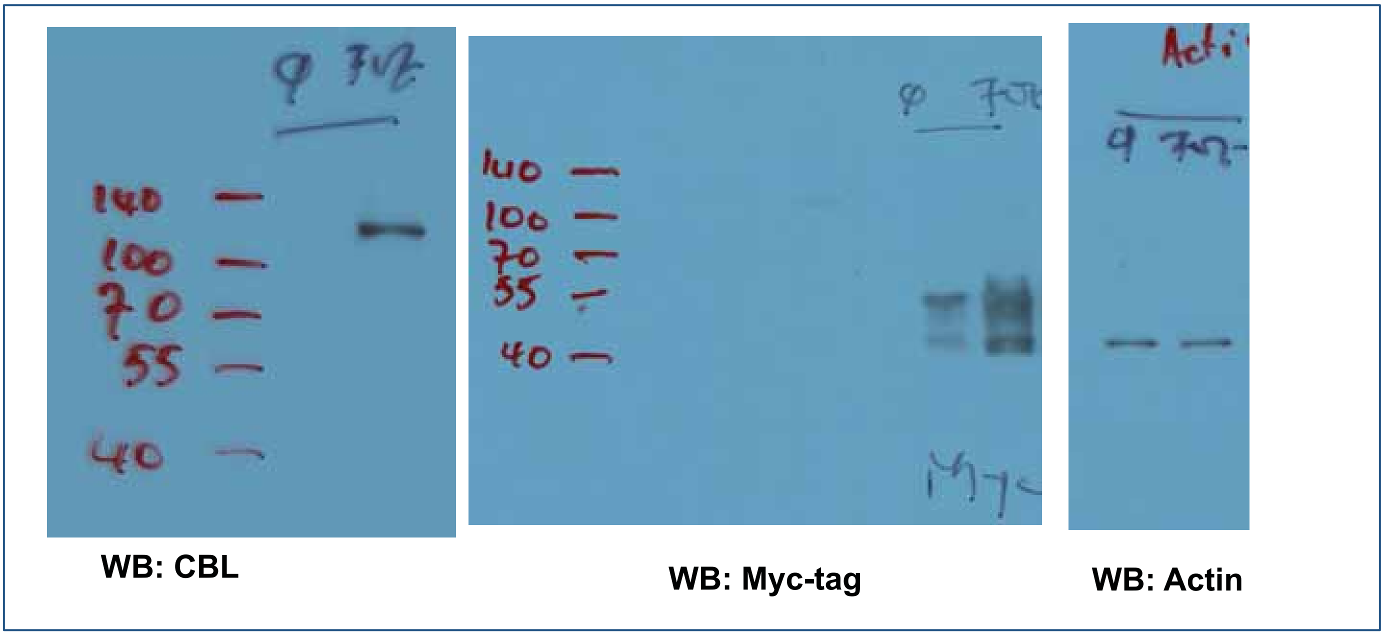

Figure 6F

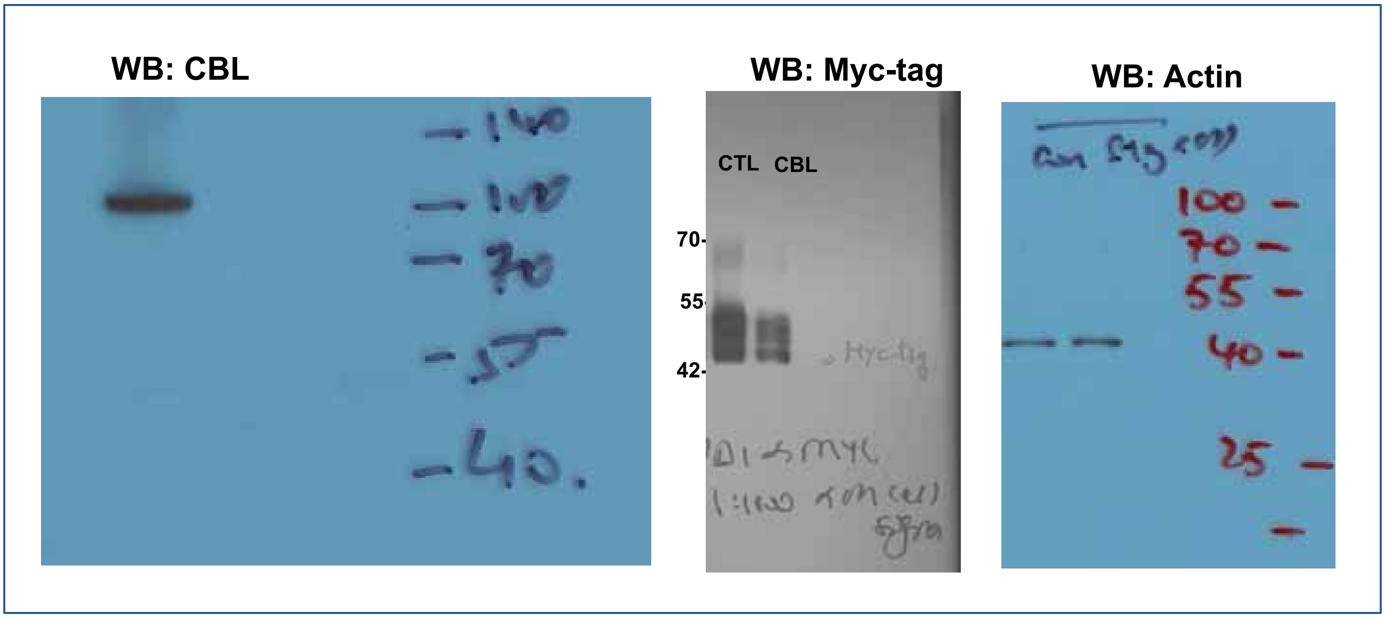

Figure 7A

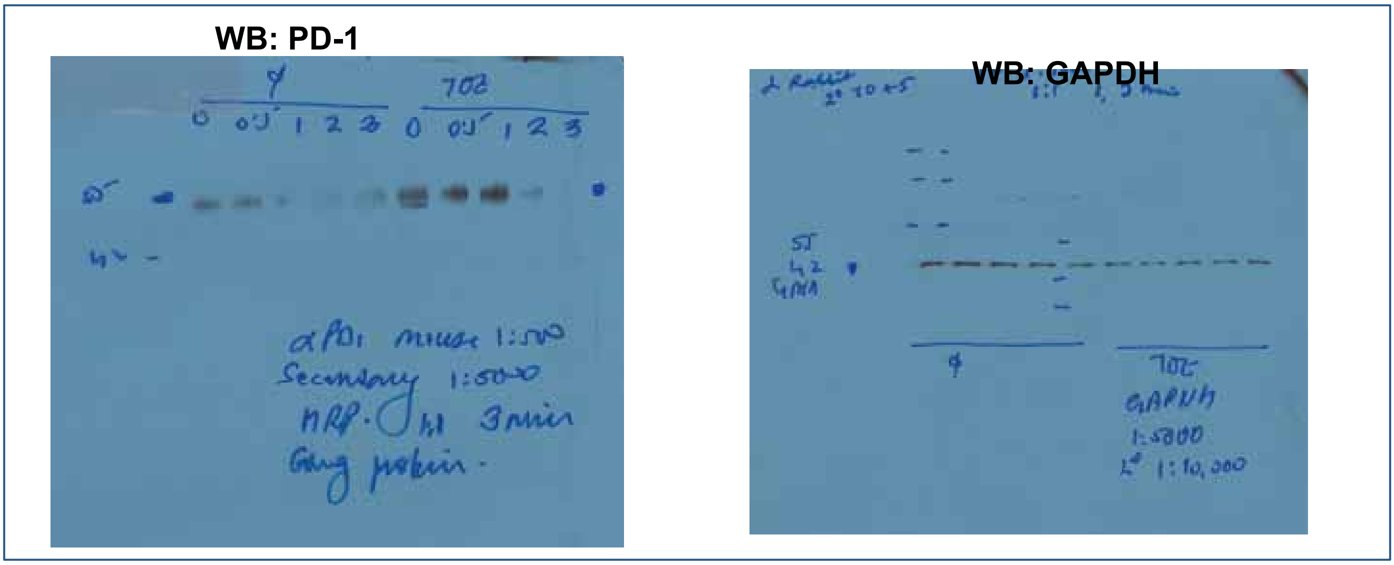

Figure 7C

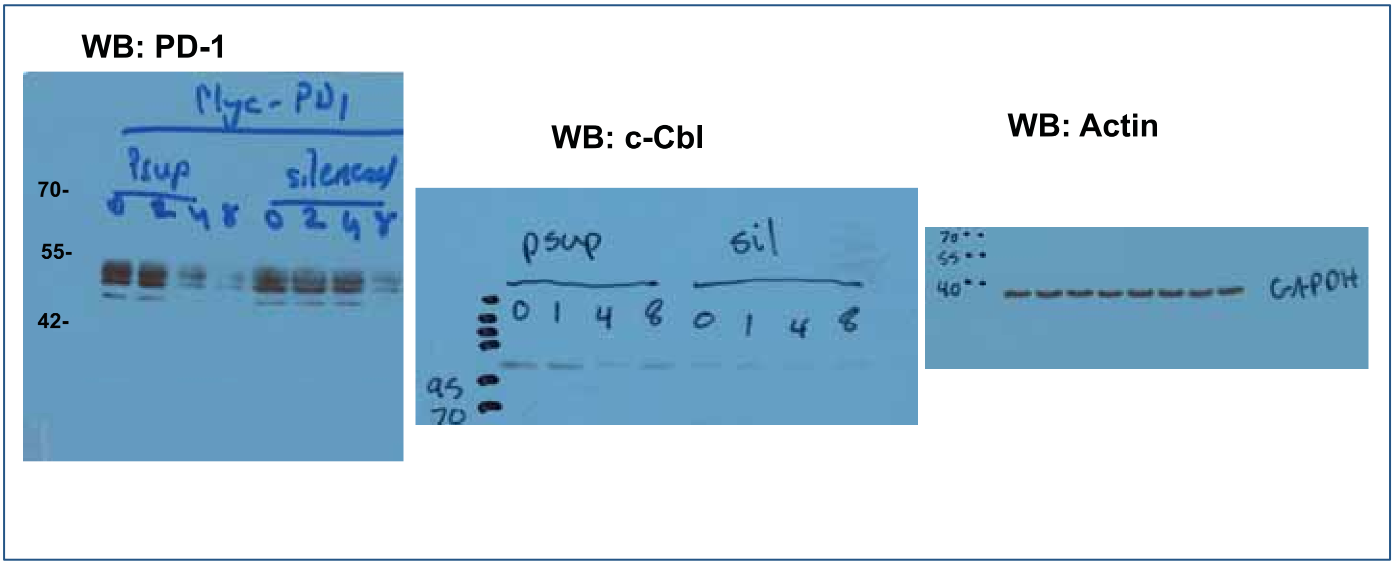

Figure 7E

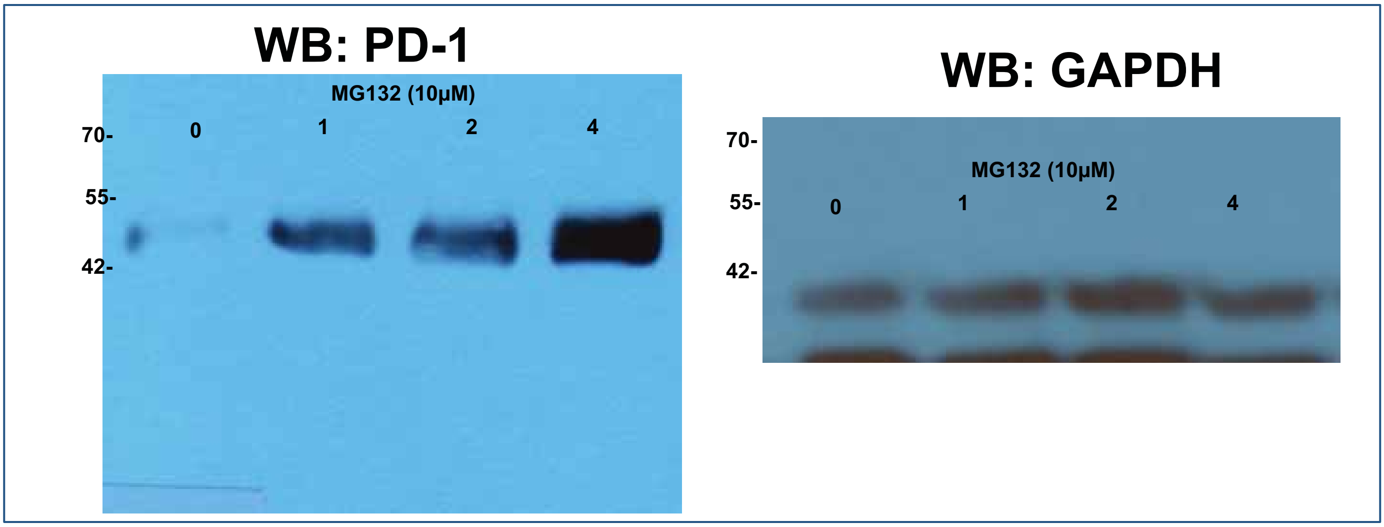

Figure 7F

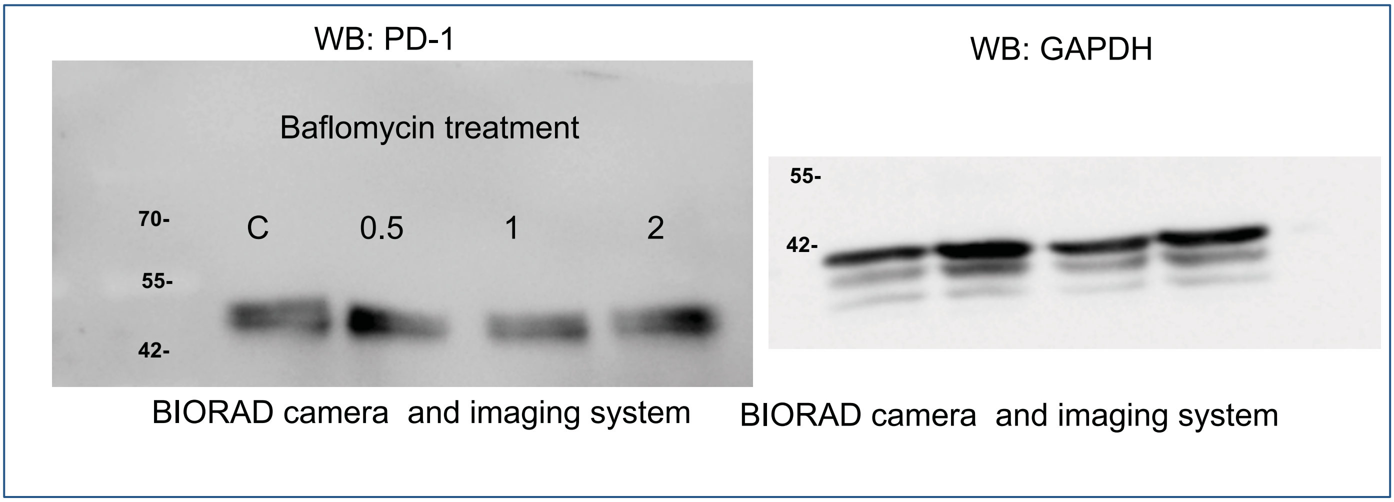

Figure 7G

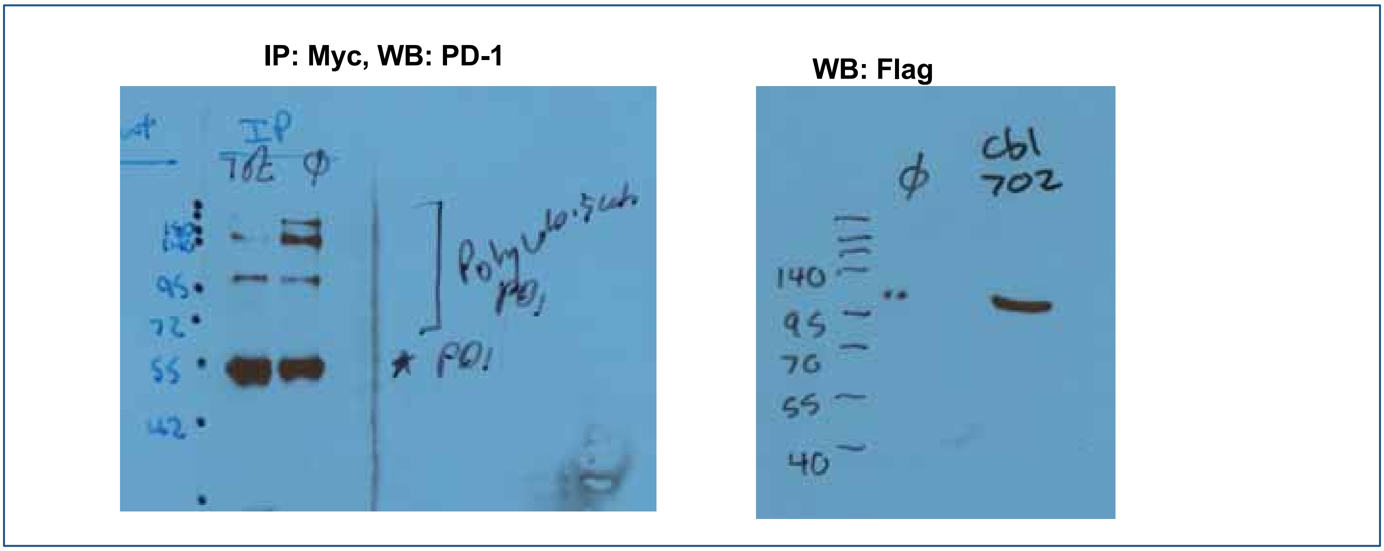

Figure 7H

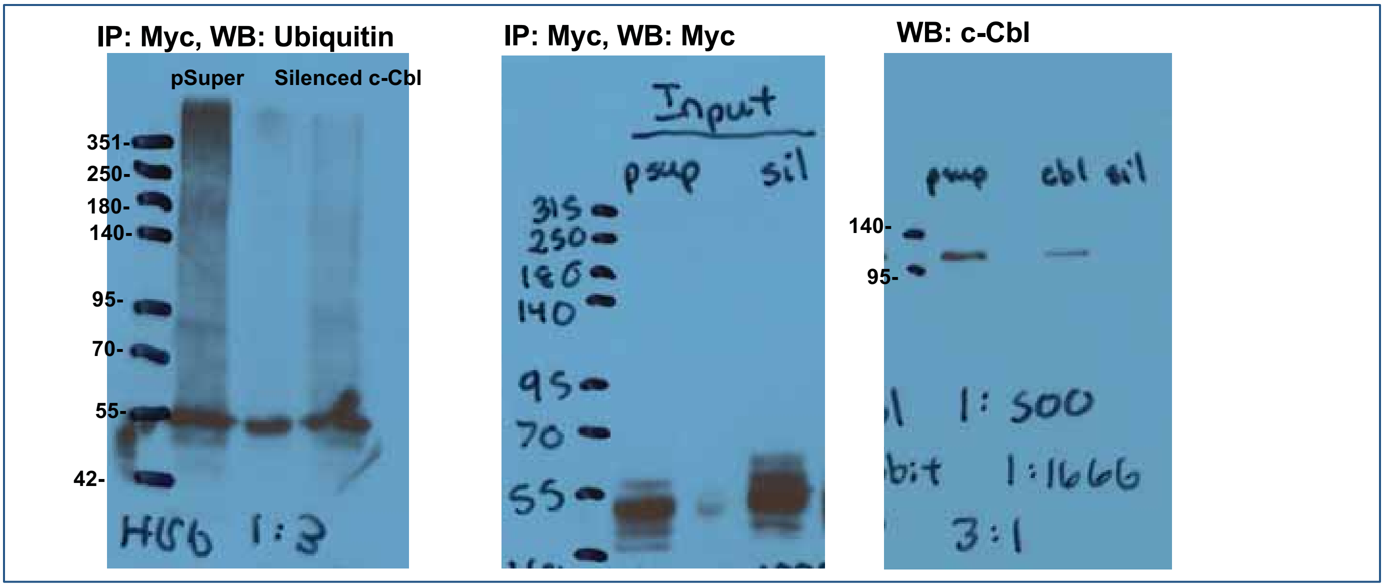

Figure 7I

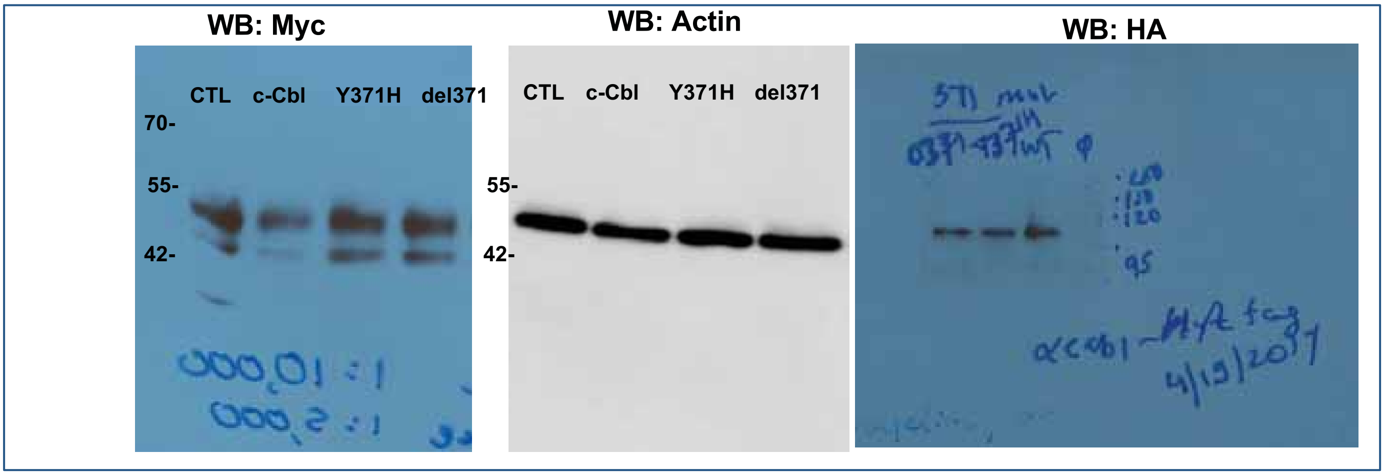

Supplementary figure 1A

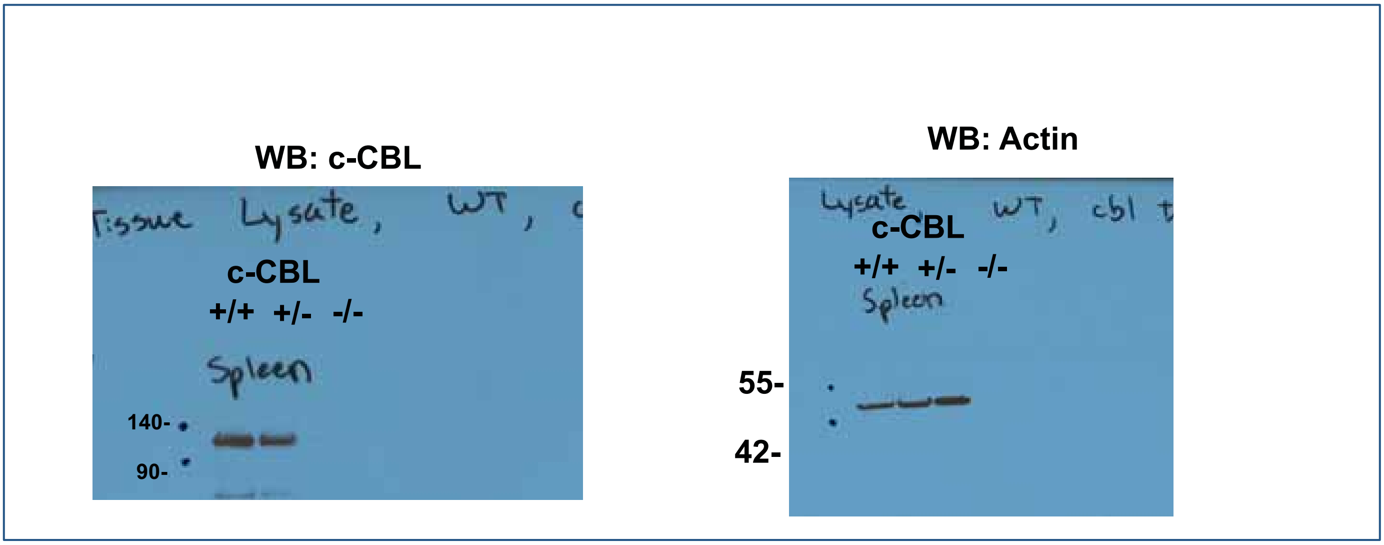

Supplementary figure 1A

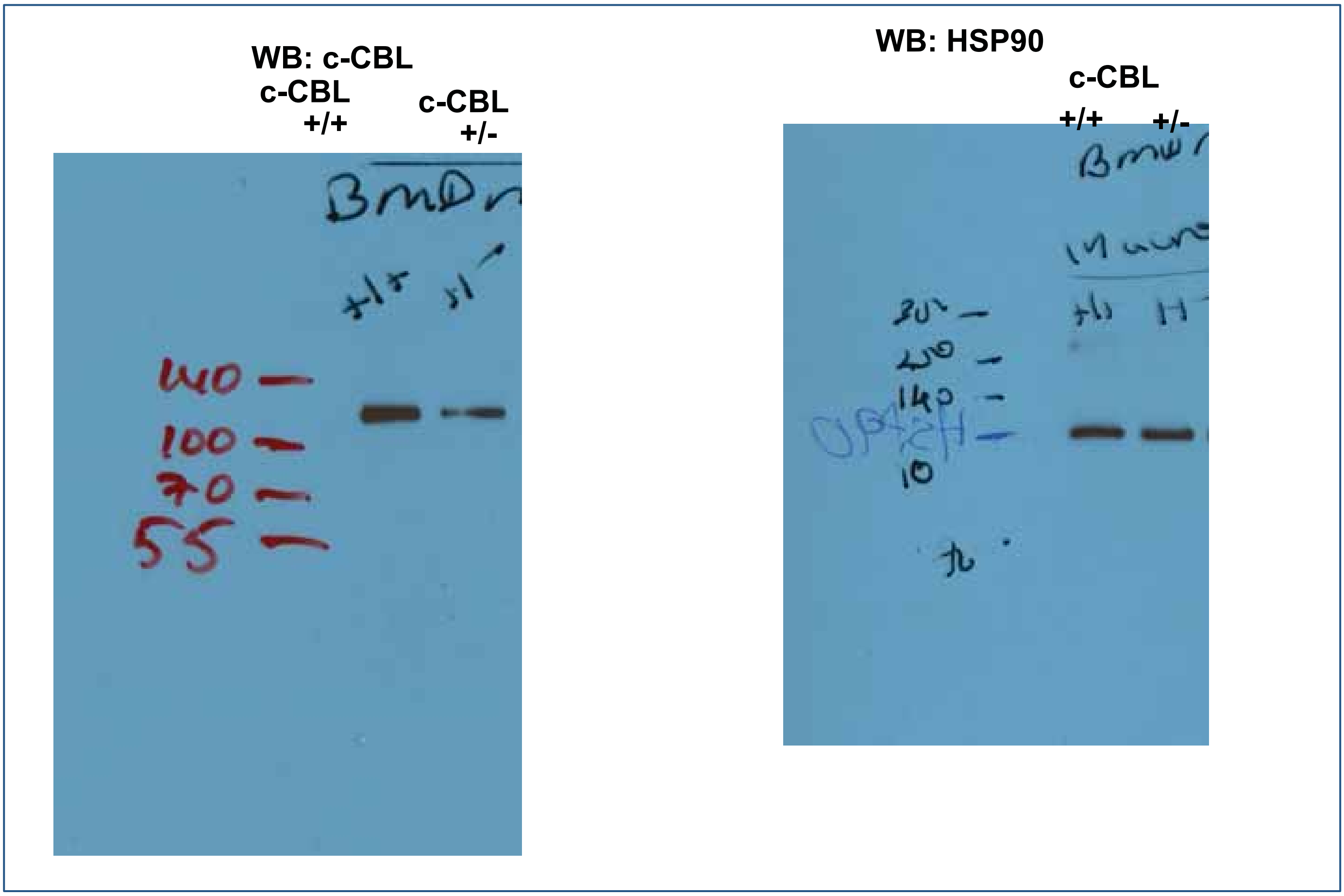

Supplement: Supplementary file 1 — Supplementary information [file 41598_2019_56208_MOESM1_ESM.pdf]
